# Determinants of Beat-to-Beat Variability of Repolarization Duration in the Canine Ventricular Myocyte: A Computational Analysis

**DOI:** 10.1371/journal.pcbi.1003202

**Published:** 2013-08-22

**Authors:** Jordi Heijman, Antonio Zaza, Daniel M. Johnson, Yoram Rudy, Ralf L. M. Peeters, Paul G. A. Volders, Ronald L. Westra

**Affiliations:** 1 Department of Knowledge Engineering, Maastricht University, Maastricht, The Netherlands; 2 Department of Cardiology, Cardiovascular Research Institute Maastricht, Maastricht University Medical Centre, Maastricht, The Netherlands; 3 Institute of Pharmacology, Faculty of Medicine, University Duisburg-Essen, Essen, Germany; 4 Department of Biotechnology and Biosciences, University of Milano-Bicocca, Milano, Italy; 5 Cardiac Bioelectricity and Arrhythmia Center, Washington University in St. Louis, St. Louis, Missouri, United States of America; University of California San Diego, United States of America

## Abstract

Beat-to-beat variability of repolarization duration (BVR) is an intrinsic characteristic of cardiac function and a better marker of proarrhythmia than repolarization prolongation alone. The ionic mechanisms underlying baseline BVR in physiological conditions, its rate dependence, and the factors contributing to increased BVR in pathologies remain incompletely understood. Here, we employed computer modeling to provide novel insights into the subcellular mechanisms of BVR under physiological conditions and during simulated drug-induced repolarization prolongation, mimicking long-QT syndromes type 1, 2, and 3. We developed stochastic implementations of 13 major ionic currents and fluxes in a model of canine ventricular-myocyte electrophysiology. Combined stochastic gating of these components resulted in short- and long-term variability, consistent with experimental data from isolated canine ventricular myocytes. The model indicated that the magnitude of stochastic fluctuations is rate dependent due to the rate dependence of action-potential (AP) duration (APD). This process (the “active” component) and the intrinsic nonlinear relationship between membrane current and APD (“intrinsic component”) contribute to the rate dependence of BVR. We identified a major role in physiological BVR for stochastic gating of the persistent Na^+^ current (I_Na_) and rapidly activating delayed-rectifier K^+^ current (I_Kr_). Inhibition of I_Kr_ or augmentation of I_Na_ significantly increased BVR, whereas subsequent β-adrenergic receptor stimulation reduced it, similar to experimental findings in isolated myocytes. In contrast, β-adrenergic stimulation increased BVR in simulated long-QT syndrome type 1. In addition to stochastic channel gating, AP morphology, APD, and beat-to-beat variations in Ca^2+^ were found to modulate single-cell BVR. Cell-to-cell coupling decreased BVR and this was more pronounced when a model cell with increased BVR was coupled to a model cell with normal BVR. In conclusion, our results provide new insights into the ionic mechanisms underlying BVR and suggest that BVR reflects multiple potentially proarrhythmic parameters, including increased ion-channel stochasticity, prolonged APD, and abnormal Ca^2+^ handling.

## Author Summary

Every heartbeat has an electrical recovery (repolarization) interval that varies in duration from beat to beat. Excessive beat-to-beat variability of repolarization duration has been shown to be a risk marker of potentially fatal heart-rhythm disorders, but the contributing subcellular mechanisms remain incompletely understood. Computational models have greatly enhanced our understanding of several basic electrophysiological mechanisms. We developed a detailed computer model of the ventricular myocyte that can simulate beat-to-beat changes in repolarization duration by taking into account stochastic changes in the opening and closing of individual ion channels responsible for all main ion currents. The model accurately reproduced experimental data from isolated myocytes under both physiological and pathological conditions. Using the model, we identified several mechanisms contributing to repolarization variability, including stochastic gating of ion channels, duration and morphology of the repolarization phase, and intracellular calcium handling, thereby providing insights into its basis as a proarrhythmic marker. Our computer model provides a detailed framework to study the dynamics of cardiac electrophysiology and arrhythmias.

## Introduction

Beat-to-beat variability of repolarization duration (BVR) is an intrinsic characteristic of cardiac function that can be observed at multiple scales, from temporal variations in action-potential (AP) duration (APD) of the single cardiac myocyte to instability of the QT interval on the body-surface ECG [1]–[3]. When increased by adverse repolarization changes, it is a better marker of proarrhythmia than repolarization prolongation *per se* in various experimental models of torsades-de-pointes ventricular tachycardia [4]–[6] and in human cardiac pathologies [2], [7]. Recently, we reported an important rate-dependent role for abnormal Ca^2+^ handling and the slowly activating delayed-rectifier K^+^ current (I_Ks_) in the increased BVR observed during β-adrenergic stimulation in single canine ventricular myocytes [8]. However, the exact mechanisms underlying BVR and its rate dependence under physiological conditions, as well as the various factors contributing to exaggerated BVR in pathological conditions, remain incompletely understood.

Computational models of cardiac myocyte electrophysiology have a rich history, dating back more than 50 years [9]. Recent models have provided detailed descriptions of various cardiac cell types in different species. They have created insight into the role of the different ion channels in rate-dependent alterations in repolarization, have helped to elucidate arrhythmogenic mechanisms in various pathological conditions, and have facilitated analysis of the integration of regulatory pathways and electrophysiological responses (reviewed in [10]–[12]). However, to date most computational models are deterministic and have an APD that converges to a fixed steady state or a limit cycle (fixed sequence of APDs; e.g., APD alternans) for a given pacing cycle length (CL). As such, these models are unsuitable for the study of BVR.

Tanskanen et al. [13] were among the first to investigate the effect of stochastic properties of local-control Ca^2+^ models on ventricular repolarization. They showed that a variable occurrence of arrhythmogenic early afterdepolarizations (EADs) could be explained by the stochastic gating of the L-type Ca^2+^ (I_CaL_) channel. In contrast, Sato et al. [14] described temporal repolarization variability due to the chaotic occurrence of I_CaL_-mediated EADs in a deterministic model of the H_2_O_2_-treated rabbit ventricular myocyte. These authors provided strong evidence that noise-induced transitions between states were insufficient to account for the large APD fluctuations observed under their experimental conditions. Instead, these transitions were intrinsically chaotic, although stochastic fluctuations could potentiate the complexity of the dynamics [15]. However, none of these studies addressed BVR under physiological conditions or under pathological conditions in the absence of EADs. Recently, the role of stochastic ion-channel gating in repolarization variability under physiological conditions has been described in computational studies by Lemay et al. [16] and Pueyo et al. [17]. These authors found that stochastic gating of selected ion channels, notably (late) I_Na_ and I_Ks_, could affect global BVR, quantified as the coefficient of APD variability.

A detailed investigation of the contribution of all major ionic processes to BVR in physiological and pathological conditions, including a quantitative comparison of short-term variability (STV; which includes differences between consecutive APs) to experimental data, has not yet been performed and was the aim of this study. We developed a stochastic version of our recently published model of the canine ventricular myocyte including β-adrenergic stimulation [18]. Stochastic formulations of 13 major ion channels and active ion transporters were included, and APD dynamics were compared to results obtained in isolated canine ventricular myocytes. We employed the model to obtain novel insights into the quantitative contribution of individual electrophysiological processes to cellular BVR under physiological conditions and the factors contributing to increased BVR during pathological conditions.

## Results

### Stochastic channel gating contributes to BVR

**Figure 1:**
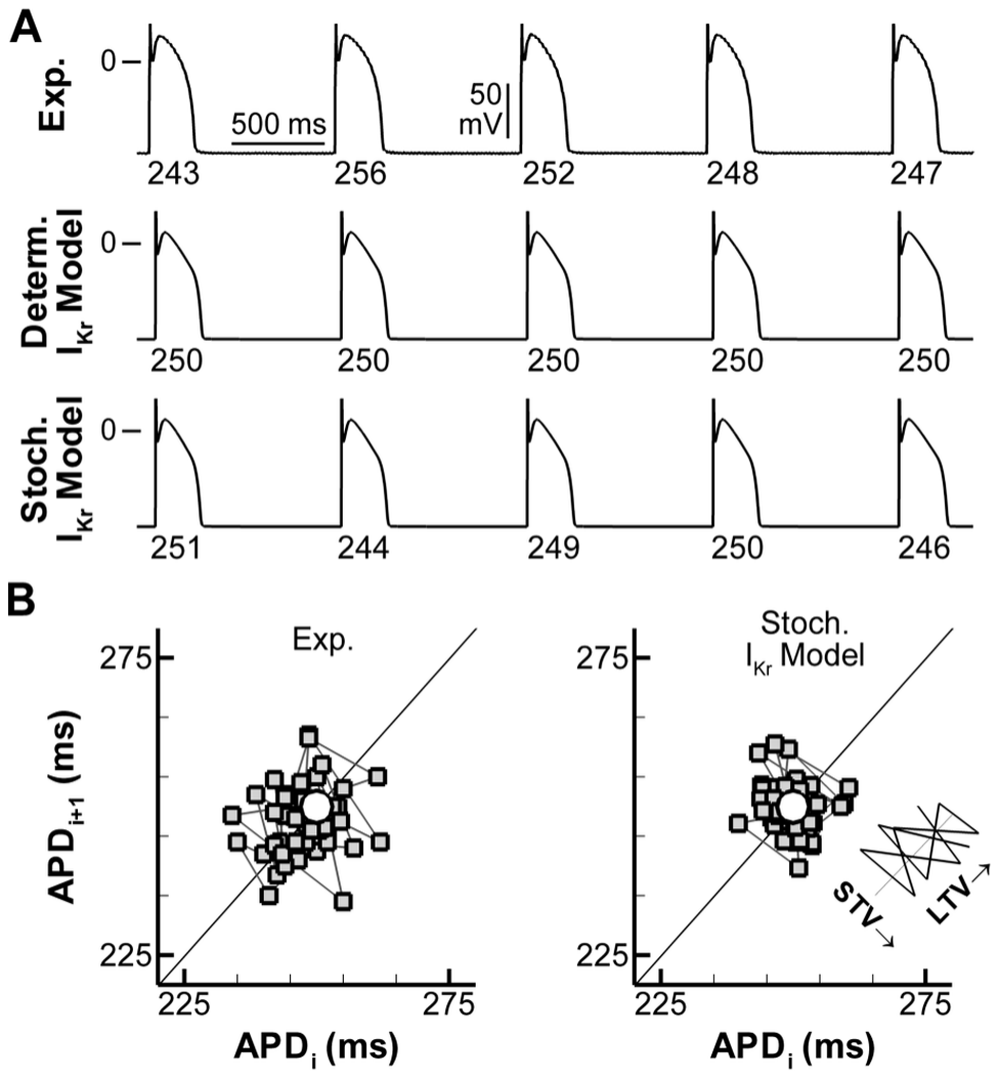
Stochastic channel gating contributes to BVR in a computational model of the canine ventricular myocyte. **A.** 5 consecutive APs in a representative canine ventricular midmyocardial cell, the deterministic computational model, or the model with a stochastic Markov formulation of I_Kr_ (top to bottom) at 1000-ms pacing CL. APD (ms) is indicated below each beat. **B.** Poincaré plot of 45 consecutive APDs for the conditions in (A). The white circle in each panel indicates the steady-state APD of the deterministic model.

AP recordings from isolated canine ventricular myocytes showed beat-to-beat variability in APD (**Figure 1A**, top panel) consistent with previous reports [1], [8]. In contrast, under physiological conditions, APD in the deterministic model (an extension of the Hund-Rudy model of the canine ventricular myocyte [19], incorporating localized β-adrenergic signaling pathways, as described by Heijman et al. [18]) converged to a steady state without APD variability (**Figure 1A**, second panel). In previous research, stochastic processes were simulated using either stochastic differential equations [17], [20], or by simulating stochastic state transitions (channel gating) in the Markov models of various ion channels [13], [21], [22]. Application of both methodologies to the Markov model of I_Kr_ resulted in APD variability (**Figure 1A**, third and fourth panel). However, these two approaches showed different temporal dynamics (**Figure 1B**). Poincaré plots of APD_i+1_ versus APD_i_ have a circular shape under these conditions in experimental recordings and in simulations with stochastic channel gating, indicating similar magnitudes of short- (STV) and long-term (LTV) variability (STV or LTV = average distance perpendicular to or along the line of identity, respectively; **Figure 1B**, inset). In contrast, BVR in simulations employing stochastic differential equations of gating variables was predominantly long term, resulting in a STV-to-LTV ratio that was markedly different from experimental recordings.

### Ionic contributors to BVR in physiological conditions

**Figure 2:**
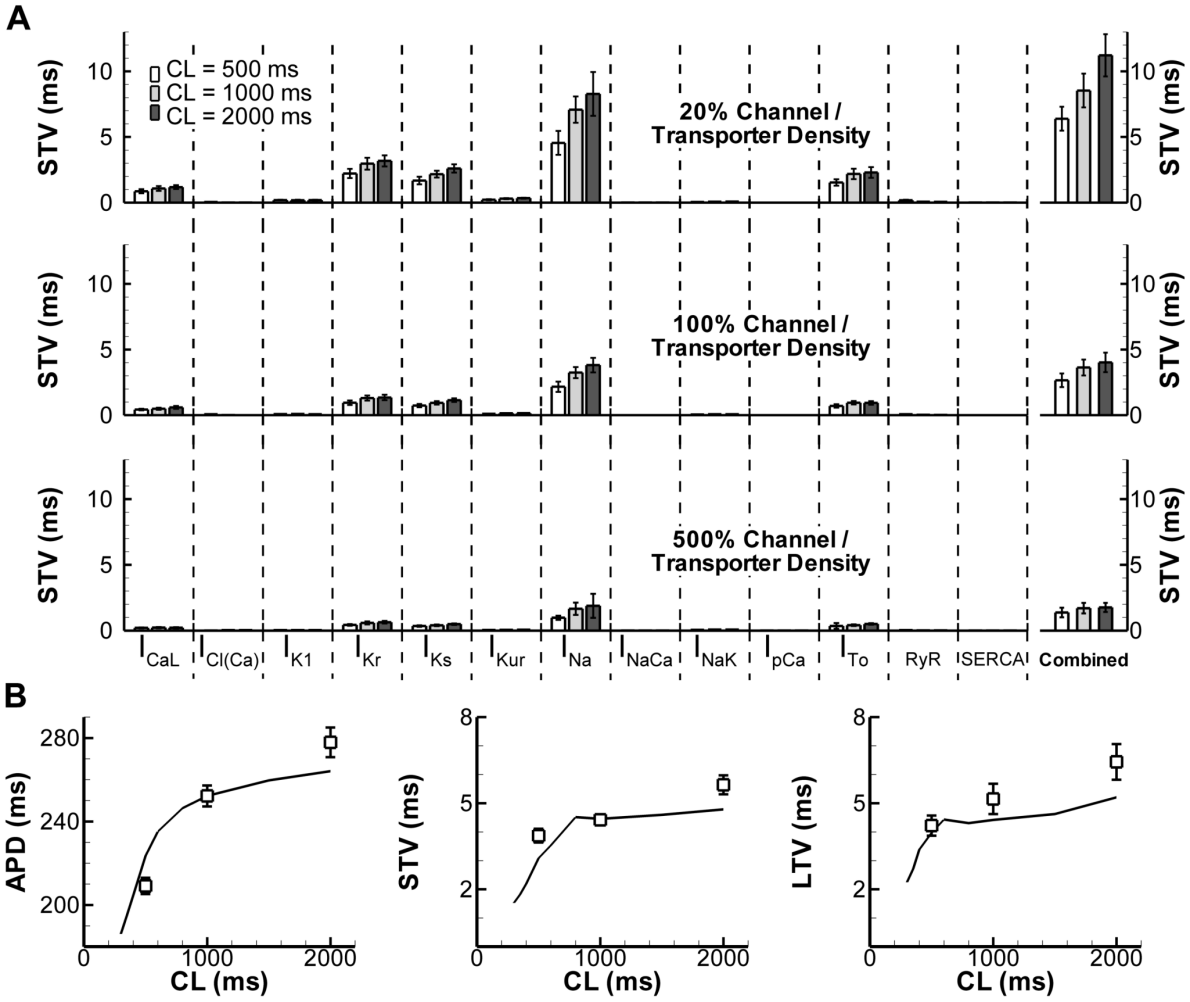
Contribution of channel density of stochastic ion currents to BVR and its rate dependence. **A.** STV magnitude induced by stochastic channel gating of individual currents in an otherwise deterministic model or stochastic channel gating of all 13 currents/fluxes combined (right-most bars) at CL of 500 ms, 1000 ms, or 2000 ms. Top panel shows 5-fold reduction in channel density (with 5-fold increase in single-channel conductance), middle panel shows channel density based on estimates from experimental data (Section 2.5 in the Supplemental Information), and bottom panel shows 5-fold increase in channel density with reduced single-channel conductance. **B.** Rate dependence of average APD (left), STV (middle) and LTV (right) in experiments (symbols) and model (lines) with stochastic gating of all 13 targets combined at 100% channel density.

To obtain insight into the direct contribution of the stochastic gating of ion currents/fluxes to whole-cell BVR, we performed simulations with stochastic formulations of each individual channel/pump/transporter in an otherwise deterministic model at CL of 500, 1000, and 2000 ms (**Figure 2A**). The number of channels underlying each current was estimated based on experimentally obtained single-channel conductance and whole-cell conductance in the model (see Section 2.5 of Text S1). To investigate the sensitivity of this parameter, we simulated normal channel density as well as a 5-fold increase or decrease in channel density (offset by a reciprocal change in single-channel conductance to maintain the same total current). A lower channel density (with larger single-channel conductance) resulted in a larger STV for all stochastic simulations. A large difference between the impacts of individual ion currents on BVR could be observed, with the largest contributions by persistent I_Na_ and I_Kr_ to STV under these conditions (**Figure 2A**). Stochastic gating of I_Ks_ also had a substantial impact on BVR, despite its small effect on APD under basal conditions in isolated myocytes [8], [23], consistent with results by Pueyo et al [17]. In contrast, pumps and exchangers, which have relatively low individual throughput but high expression density [24], contributed little to BVR. In general, BVR increased with increasing CL. When all 13 stochastic components were included, STV was larger than that obtained with any individual stochastic formulation, but the results were not additive, indicating that certain stochastic fluctuations cancel each other out. The stochastic model showed APD and BVR rate dependence similar to that observed in canine ventricular myocytes (**Figure 2B**), indicating that stochastic channel gating (particularly of I_Na_ and I_Kr_ channels) is a major contributor to the baseline BVR observed in physiological conditions.

**Figure 3:**
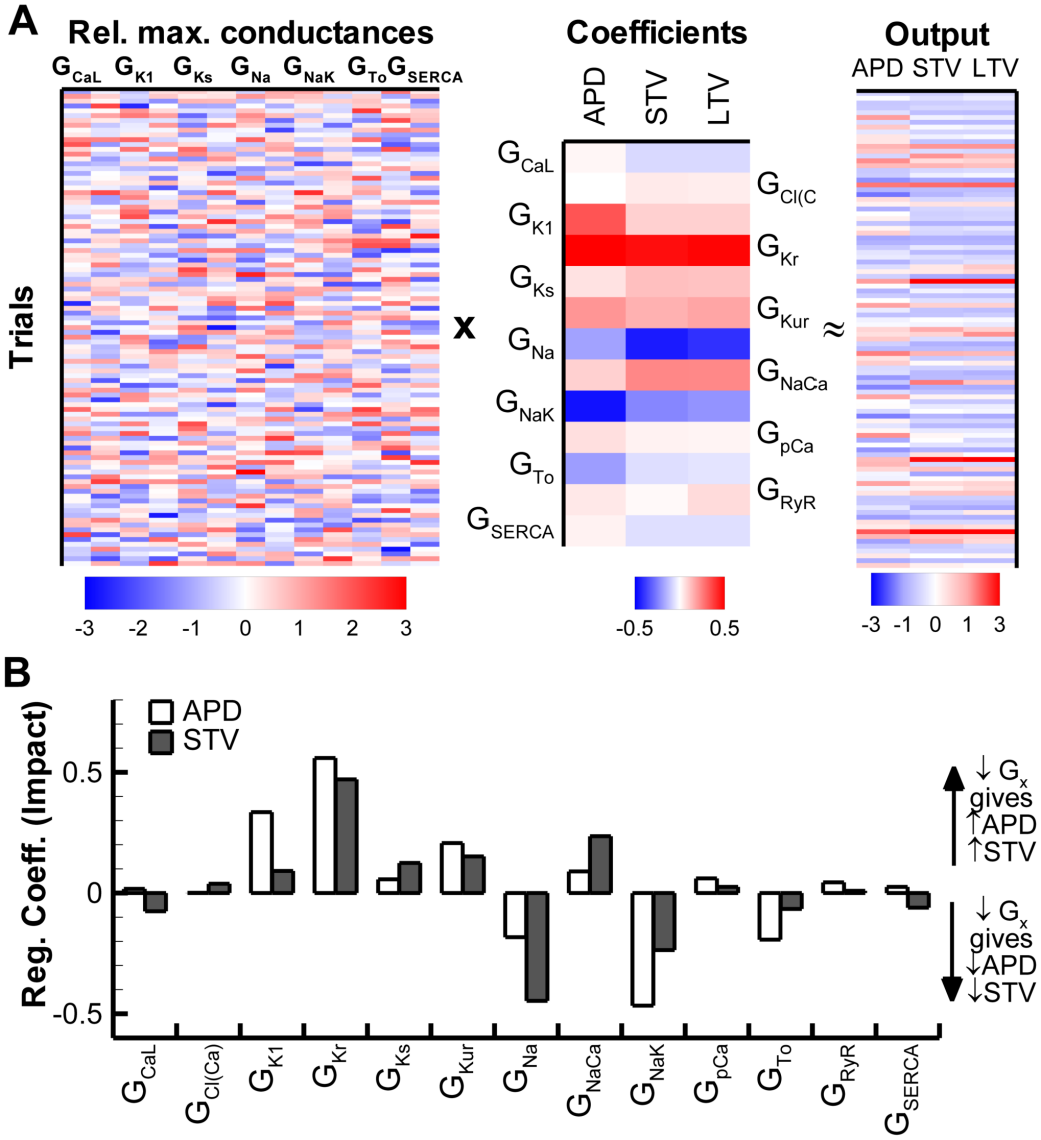
Contribution of currents to BVR determined via linear regression of 200 unique virtual myocytes. **A.** Relative changes in the maximal conductance (G_x_) of the 13 currents/fluxes (lanes correspond to the column pairs in panel B) for 100 (out of 200) trials (left panel) and corresponding changes in outputs (APD, STV and LTV) during steady-state pacing at CL = 1000 ms (right panel). Middle panel shows the coefficients that indicate the contribution of each current to every output measure as determined via linear regression. **B.** Bar plot of the magnitude of the coefficients from panel A regarding their impact on APD (white bars) or STV (shaded bars). I_Kr_ and I_Na_ have a large impact on both APD and BVR, consistent with the results from **Figure 2**. In addition, I_NaK_ also strongly affects STV. LTV showed similar pattern as STV and is not shown for clarity.

In addition to a direct impact on V_m_ fluctuations, individual ion channels may modulate STV indirectly (e.g., via fluctuations in intracellular ion concentrations that affect other currents). To dissect the effect of channel stochasticity versus effects of maximal conductance on BVR in the fully stochastic model, we employed the linear-regression method proposed by Sobie ([25], [26] and in Section 3 of Text S1). Although the linear regression is an approximation of a strongly non-linear system, this approach has previously been employed to study the contribution of different ionic currents to various pathophysiological processes [25], [26]. We simulated 200 parameter sets in which the maximal conductance of each of the currents was scaled based on a Gaussian distribution with mean 1.0 and standard deviation (Std) 0.3. For each parameter set, mean APD, STV, and LTV were determined at steady state during stochastic simulations at 1000-ms CL (**Figure 3**). The contribution of each current was determined by performing a linear regression on the parameter settings (maximal conductances of individual currents) and output measures (**Figure 3A**). Consistent with the results based on the direct stochastic impact shown in **Figure 2**, the linear regression analysis identified major roles for alterations in conductances of I_Na_ and I_Kr_ in modulating both APD and STV (**Figure 3B**). In addition, this approach also identified a substantial impact of I_NaK_ and I_NaCa_ on STV. Because the stochastic gating of these currents did not result in significant BVR when simulated in an otherwise deterministic model, it follows that variations in the maximal conductance of these targets affect STV via other parameters (e.g., APD, intracellular ion concentrations, etc.), which remains to be confirmed experimentally.

**Figure 4:**
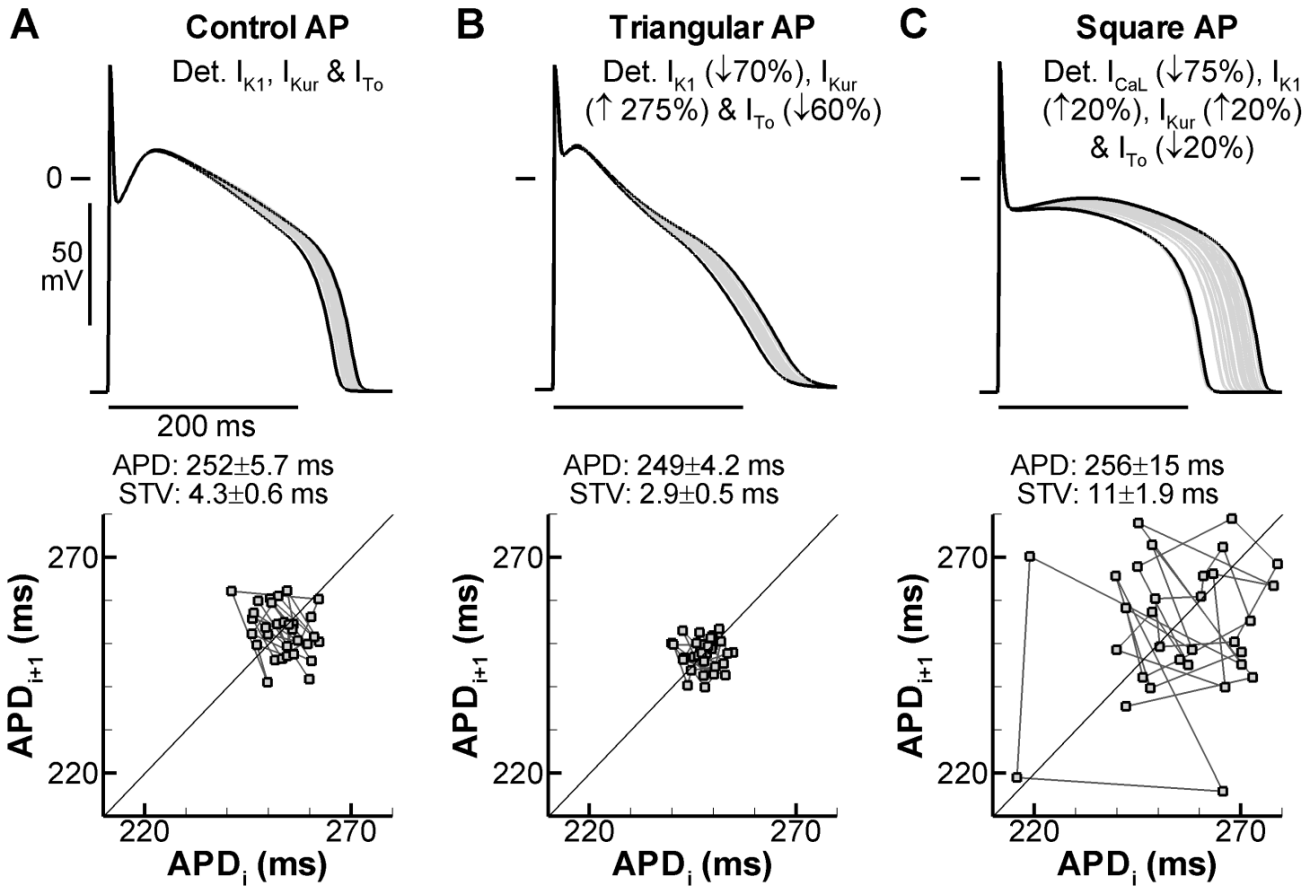
Effect of AP morphology on BVR. **A.** Overlay of 30 APs (top panel) and Poincaré plot of corresponding APDs (bottom panel) for the control myocyte, without alterations in ion currents, simulated with deterministic I_CaL_, I_K1_, I_Kur_, and I_To_ and stochastic gating of the remaining 9 currents. APs with the shortest and longest duration are shown in black, others in grey. Average APD and STV are indicated below the APs. **B.** Similar to panel A for a triangular AP morphology obtained by reducing I_K1_ and I_To_ (by 70% and 60%, respectively) and increasing I_Kur_ (by 275%). **C.** Similar to panels A and B for a square AP morphology obtained by increasing I_K1_ and I_Kur_ (by 20% each) and decreasing I_CaL_ and I_To_ (by 75% and 20%), respectively.

One potential mechanism through which individual ion channels may impact BVR is their influence on AP morphology [27]. To study this, we increased or decreased the amplitude of I_K1_, I_Kur_, I_To_ and/or I_CaL_ to adjust AP morphology without affecting average APD. The three currents were simulated deterministically in all cases to prevent any direct effects of the altered current amplitudes on BVR. This protocol allowed us to compare the effect of AP morphology on the stochastic gating of the 9 remaining currents and on BVR in the absence of confounding changes in average APD. STV was 4.3±0.6 ms in the control model with deterministic I_K1_, I_Kur_, I_To_, and I_CaL_, and APD was 252±5.7 ms (**Figure 4A**). When I_K1_ and I_To_ were reduced by 70% and 60%, respectively, and I_Kur_ was increased by 275%, a triangular AP morphology was obtained with similar APD (249±4.2 ms) but significantly lower STV (2.9±0.5 ms; **Figure 4B**). Interestingly, with a square AP morphology (I_CaL_ and I_To_ reduced by 75% and 20%, respectively, I_Kur_ and I_K1_ increased by 20%), variability was strongly increased (STV = 11±1.9 ms; **Figure 4C**) compared with control AP morphology. This pattern suggests that, in addition to APD, AP morphology can strongly affect BVR, whereby a conspicuous AP plateau is associated with increased BVR.

### Effects of cell-to-cell coupling on BVR

**Figure 5:**
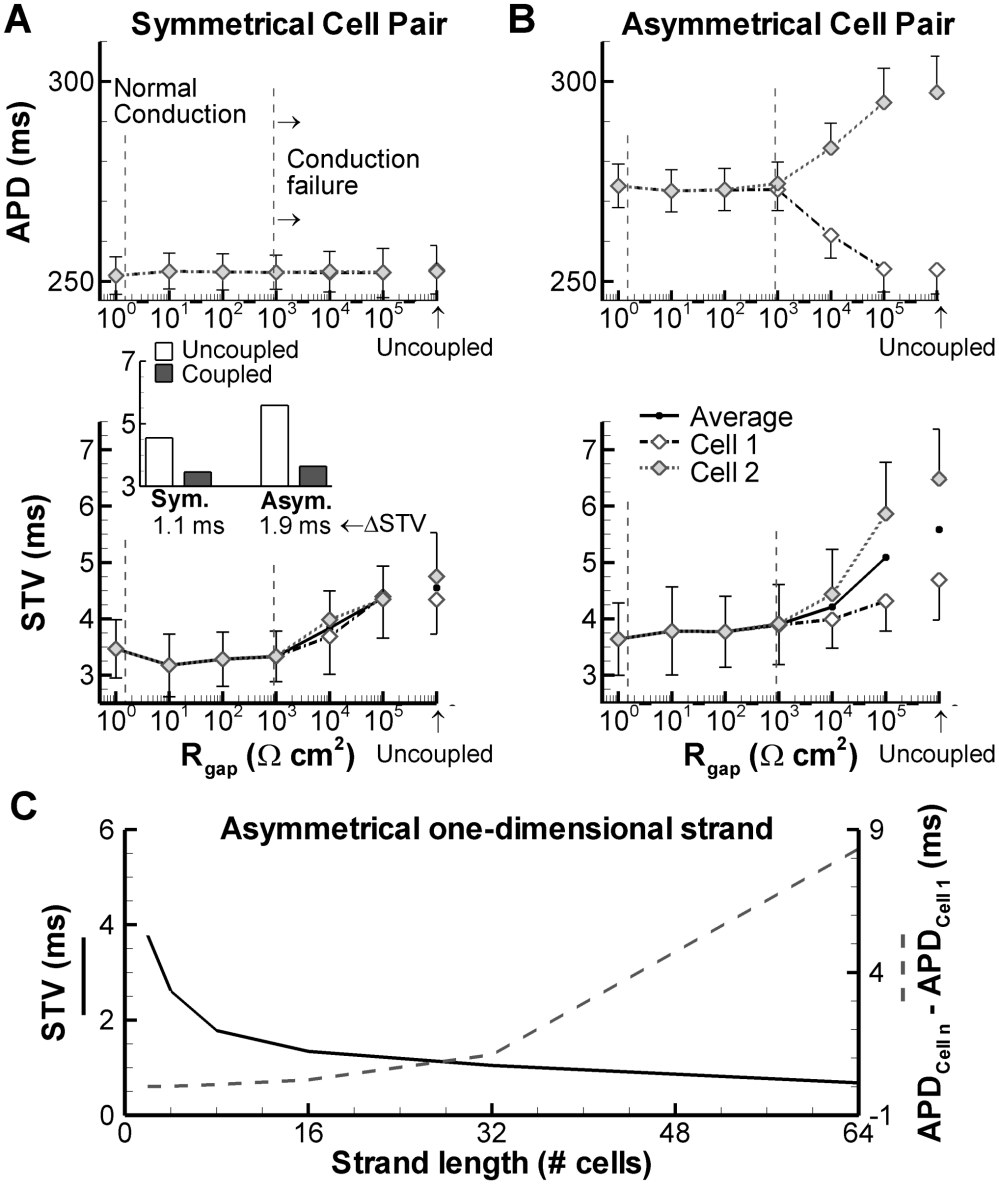
Effect of cell-to-cell coupling on BVR. **A.** APD (top panel) and STV (bottom panel) of two identical cells for various degrees of electrical coupling. Normal coupling (left vertical dashed line) and critical coupling for successful conduction in a one-dimensional strand of virtual myocytes (right vertical dashed line) are indicated. Both cells received external stimulation. **B.** Similar to panel A for two cells of which one is prolonged via current injection (−0.1 pA/pF). Cell-to-cell coupling causes a mild decrease in average STV (1.1 ms) that is more pronounced in the case of an asymmetrical cell pair (1.9 ms; inset). **C.** Effect of strand length on temporal (solid line) and spatial (dashed line) dispersion of repolarization in an asymmetrical one-dimensional strand. Half of the strand received additional current injection to prolong APD, similar to panel B. BVR decreased with increasing strand length, whereas spatial dispersion of repolarization increased for longer strands.

Zaniboni et al. [1] have previously shown that the electrical coupling of two myocytes reduced their temporal repolarization variability, which was confirmed in the modeling study by Lemay et al [16]. When identical cells were coupled in our simulations, the overall temporal variability (coefficient of variance: 100%×Std(APD)/mean(APD)) in the model was reduced from 2.4% to 1.9%, quantitatively similar to that observed by Zaniboni et al. in guinea-pig ventricular myocytes (2.3±1.2% in uncoupled cells vs. 1.5±0.6% in cell pairs). We observed a reduction in BVR of 1.1 ms in cell pairs compared to uncoupled cells when two identical cells were coupled (**Figure 5A**). Gap-junction conductance did not influence BVR over the range of values that would result in successful propagation in a one-dimensional strand [28]. Interestingly, Zaniboni et al. also reported that there was an asymmetrical redistribution of APD when a cell with a long APD was coupled to a cell with short APD, by which the long APD shortened more than the short APD prolonged. We hypothesized that this asymmetrical response may also apply to BVR and prolonged APD in one of the two cells through the injection of a constant, deterministic current for the duration of the APD (**Figure 5B**). BVR was larger in the cell with prolonged APD, thereby increasing the average BVR. When the two cells were coupled, spatial APD dispersion was lost. Although BVR remained larger than that of the symmetrical cell pair, the decrease in average BVR compared with the uncoupled situation was more pronounced (1.9 ms; **Figure 5A**, inset). Moreover, BVR was further reduced (but not eliminated) when more cells were coupled together in a one-dimensional strand (**Figure 5C**), although, as expected, the spatial dispersion of repolarization increased with increasing length of the strand. Taken together, these data suggest that cell-to-cell coupling not only reduces spatial dispersion of repolarization but may also limit excessive BVR of vulnerable regions. As such, conditions in which coupling is reduced (e.g., in ischemia) may lead to increased BVR.

### BVR rate dependence

Because of the hyperbolic relationship between the rate of repolarization and APD, sensitivity to changes in net membrane current (I_m_) may be higher in longer APs, thereby contributing to reverse rate-dependence of APD modulation and, thus, to BVR [27], [29]. This mechanism, of which the physiological relevance is supported by recent experimental findings [30], has been identified as “intrinsic” because it reflects a numerical property independent from channel gating [27]. Nevertheless, the impact of stochastic channel gating on net membrane current might also be rate dependent, contributing an additional source of APD variability, which we will refer to as “active” to differentiate it from the “intrinsic” component.

We hypothesize that i) both the “intrinsic” and the “active” components may underlie BVR reverse rate-dependence (**Figure 2B**) and ii) that individual ionic conductances may contribute unequally to the “active” component. To address these hypotheses, membrane potential and net membrane current (V_m_ and I_m_) were recorded for 30 beats during steady-state stimulation at different cycle lengths. The mean and Std of I_m_ and V_m_ over these 30 beats was measured at each time point during the action potential.

**Figure 6:**
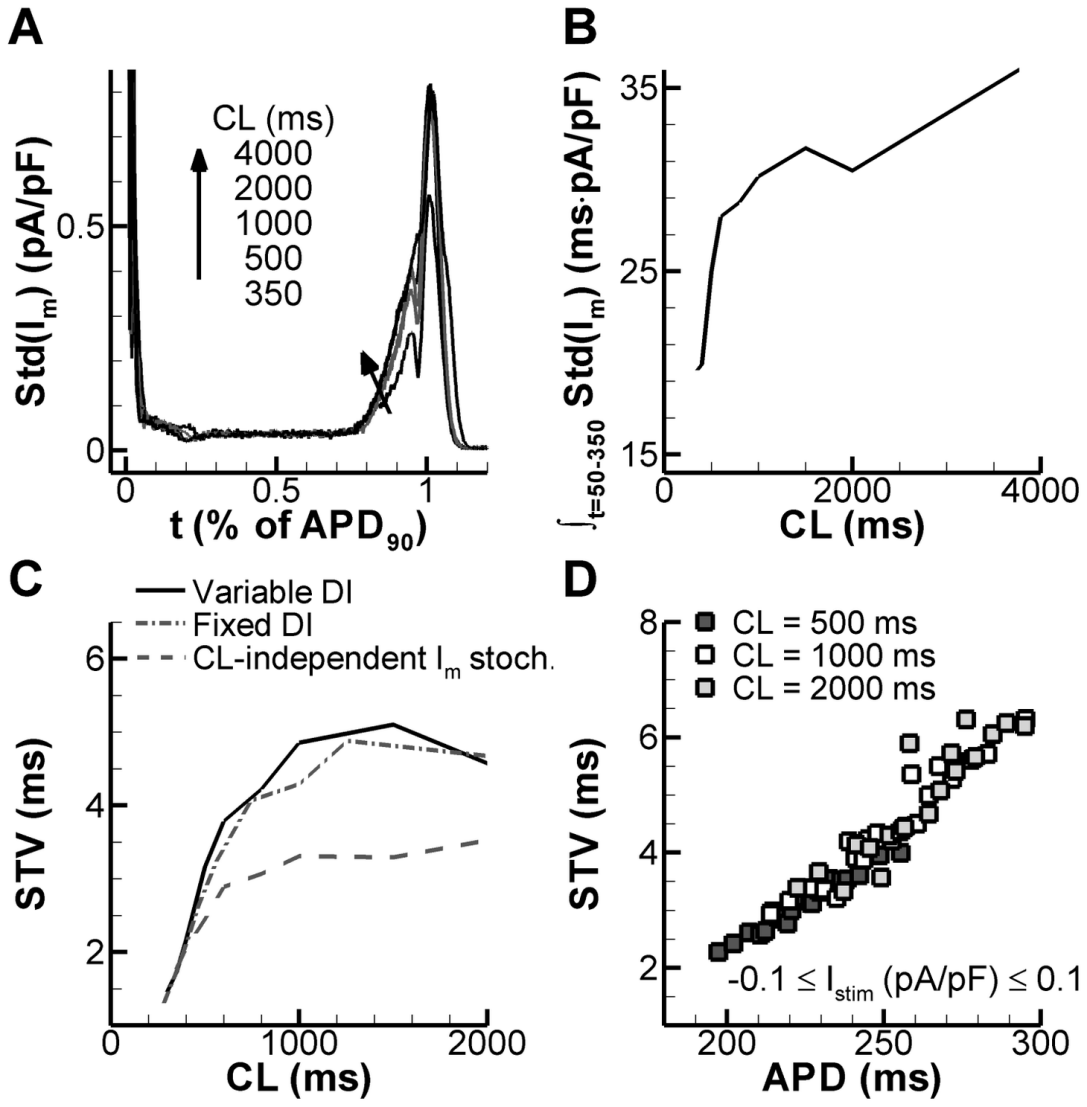
Role of APD and stochastic gating in BVR reverse rate dependence. **A.** Magnitude of channel gating stochastics (assessed by Std(I_m_) for 50 beats) over time for CL of 350–4000 ms using the fully stochastic model under control conditions. **B.** Rate dependence of total magnitude of I_m_ fluctuations (given by area under Std(I_m_) curve). **C.** STV rate dependence in the fully stochastic model during fixed-CL pacing (solid line) or fixed-DI pacing (dash-dotted line), or in the deterministic model during fixed-CL pacing with a CL-independent stochastic term (see Results, section “BVR rate dependence”) added to I_m_ (dashed line). CL-independent stochastic behavior results in a blunted STV rate dependence. **D.** STV vs. APD relationship at CLs of 500 ms (dark grey symbols), 1000 ms (white symbols), or 2000 ms (light grey symbols). APD was varied through injection of a deterministic stimulus current between −0.1 and 0.1 pA/pF for the duration of the AP.

In simulations with the stochastic model, Std(I_m_) was found to vary along the action-potential course, reaching a maximum during phase-3 repolarization (**Figure 6A**). While such a Std(I_m_) profile was present at all CLs, Std(I_m_) global magnitude increased at longer CLs (**Figure 6A,B**). The observation that Std(I_m_) is rate dependent confirms the presence of an “active” component in BVR rate dependence.

According to the “intrinsic” component concept, rate-independent Std(I_m_) should still result in rate-dependent BVR [27]. Thus, to quantify the impact of the intrinsic component on BVR rate-dependency, BVR was measured at various CLs in the presence of rate-independent Std(I_m_). To this aim, a stochastic Gaussian component was selectively added to the V_m_ update step of the deterministic model. The amplitude of the stochastic component (α = 1.5; Section 2 of Text S1) was chosen such that BVR at CL = 300 ms matched that of the fully stochastic model. As shown in **Figure 6C**, rate dependence of STV was blunted, but not eliminated by this procedure. The remaining rate dependence of STV (dashed line) reflects the “intrinsic” component contribution (i.e., even with the same amount of I_m_ “noise”, BVR is larger at longer CLs).

If affected by the “intrinsic” component only, BVR magnitude should be independent of diastolic interval (DI) [27]. However, it was less obvious whether this should apply also to overall BVR, i.e., including the “active” component. To answer this question, in the stochastic model APD was prolonged through injection of a constant current and the resulting STV vs. APD relationship was measured at three CLs (**Figure 6D**). The STV/APD relationships at the three CLs largely overlapped, indicating that mean APD, rather than DI, is also the main determinant of the “active” component. To confirm this conclusion, we adapted the pacing protocol in the simulations such that the virtual cell was paced with a fixed diastolic interval. To this end, APD was determined online (i.e., during the simulation), and the next pacing instant was set to achieve a pre-specified DI. Multiple simulations were performed with different pre-specified DIs to obtain a curve similar to that for BVR rate dependence. In fact, when BVR was plotted vs. CL (determined via CL = mean APD+DI), an identical BVR/CL relationship was obtained compared to normal pacing (compare “fixed DI” and “variable DI”; **Figure 6C**), confirming that under these physiological conditions, variations in DI do not contribute to BVR rate dependence. In contrast, in the chaotic models of Sato et al. in the setting of EADs, beat-to-beat APD differences occur because of a steep APD/diastolic interval (DI) relationship [14].

### Mechanisms contributing to exaggerated BVR in drug-induced repolarization prolongation

**Figure 7:**
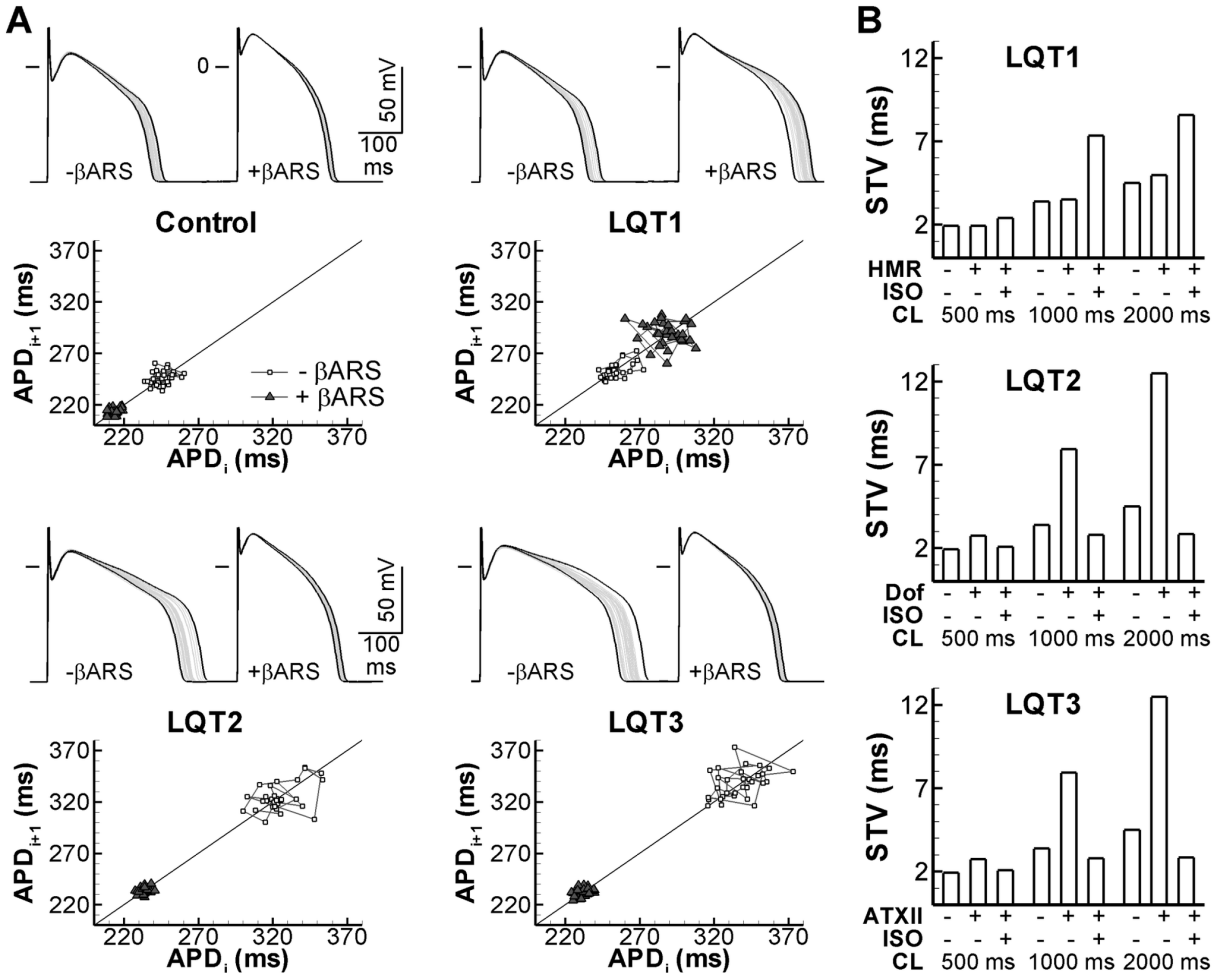
BVR in simulated LQT syndrome types 1–3 in the absence or presence of βARS. **A.** Overlay of 30 consecutive APs in the absence (−βARS) or presence (+βARS) of β-adrenergic receptor stimulation under control conditions (top-left panel) or simulated LQT1 (top-right panel), LQT2 (bottom-left panel), or LQT3 (bottom-right panel) at 1000-ms CL. Shortest and longest APs are shown in black, intermediate APs in grey. A Poincaré plot of the 30 APDs is shown below. **B.** Quantification of BVR in LQT1-3 at CL of 500, 1000, or 2000 ms in the absence or presence of βARS. HMR indicates simulation of the I_Ks_ blocker HMR1556 (simulated LQT1), Dof simulation of the I_Kr_ blocking drug dofetilide (LQT2) and ATXII indicates simulations with enhanced persistent I_Na_ (LQT3). βARS reduces BVR significantly in LQT2 and LQT3, but not in LQT1, consistent with experimental results [8].

We previously reported that BVR is increased in pharmacological models of long-QT syndrome (LQT) type 2 (using the I_Kr_-blocking drug dofetilide) and LQT3 (increased persistent I_Na_ due to ATXII) and that this BVR could be reduced by β-adrenergic stimulation (βARS) [8]. The present finding that BVR is most sensitive to I_Kr_ and I_Na_ modulation (**Figure 2**) is consistent with these observations. In contrast, we found that during I_Ks_ inhibition (using HMR1556), βARS significantly increased BVR, whereas HMR1556 alone had no effect on BVR [8]. Notably, all these experimental findings were reproduced by the model (**Figure 7A,B**), indicating that it can be employed to study the factors contributing to exaggerated BVR in pharmacological representations of LQT1-3.

**Figure 8:**
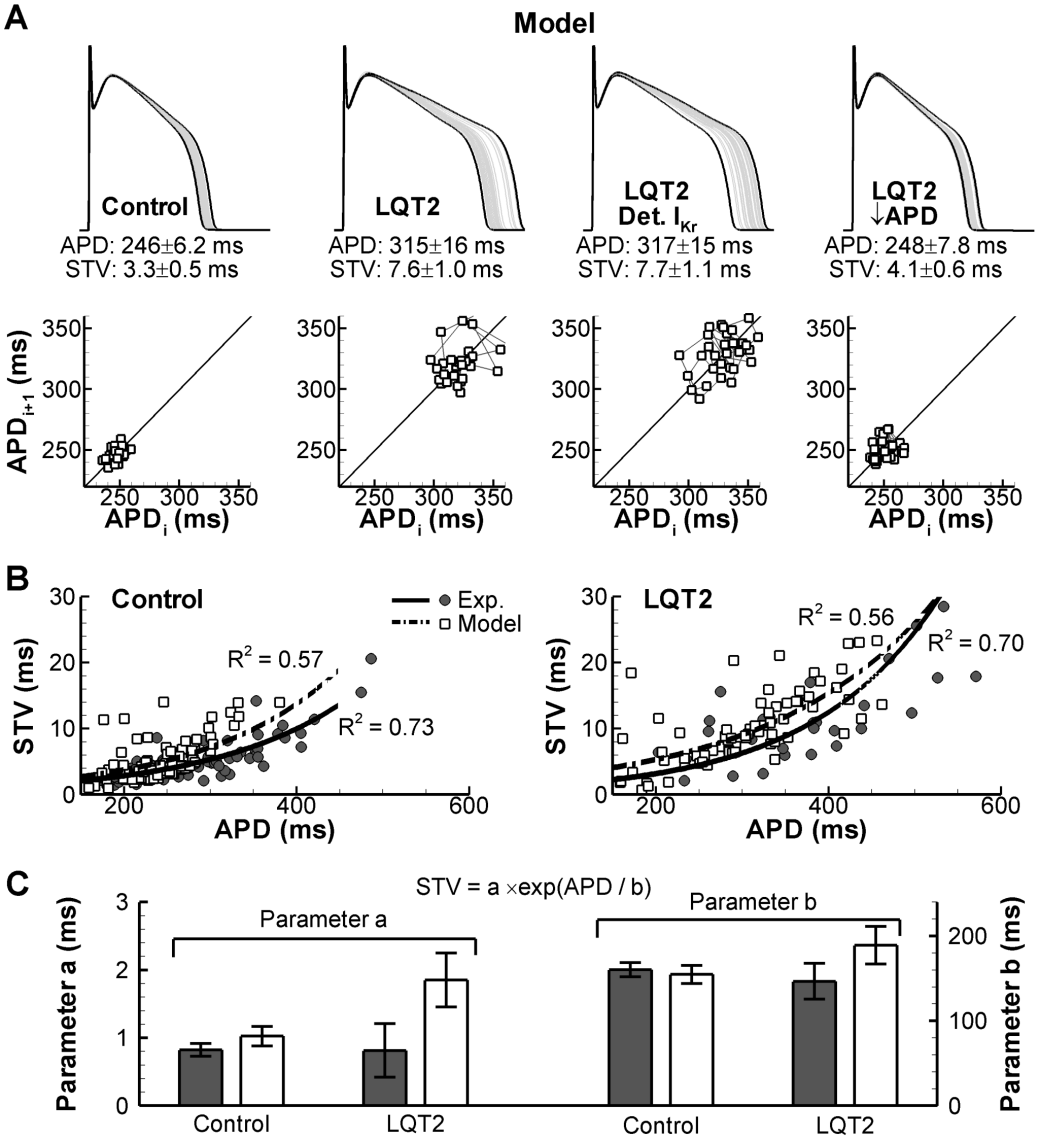
Role of APD in the observed increase in BVR under simulated LQT2 conditions. **A.** Overlay of 30 consecutive APs in the model using control conditions, simulated LQT2, simulated LQT2 with deterministic I_Kr_, or simulated LQT2 with reduced APD due to injection of a deterministic stimulus current. Shortest and longest APs are shown in black, intermediate APs in grey. APD, STV, and Poincaré plots are shown below each overlay. **B.** STV vs. APD relationship under control conditions (left panel) or LQT2 conditions (right panel) in individual canine ventricular myocytes (filled symbols) or individual model cells (open symbols; based on whole-cell conductances drawn from a Gaussian distribution, as in **Figure 3A**). Data were fit with a monoexponential function (lines). **C.** Parameters of the monoexponential fits of panel B under control and LQT2 conditions in experiments (grey bars) and model (white bars). The model shows a quantitatively similar STV vs. APD relationship as experiments, and this relationship is not different between control and LQT2 conditions.

The contribution of both intrinsic and active mechanisms to BVR rate dependence (**Figure 6**) suggests that the increased BVR observed in pharmacological models of LQT syndrome (**Figure 7**) could result directly from APD changes. On the other hand, APD and BVR changes may be dissociated under these conditions. To investigate the effect of APD prolongation on BVR, we employed a deterministic current injection to reduce average APD back to baseline levels. When APD was reduced, BVR was also reduced to control values (STV equaled 3.3±0.5, 7.6±1.0, and 4.1±0.6 ms in control, LQT2, and LQT2 with reduced APD, respectively; **Figure 8A**). In contrast, removing the stochastic gating of I_Kr_ did not significantly alter BVR (7.7±1.1 ms) compared to LQT2 simulations with stochastic I_Kr_ gating. These findings suggest that the increased BVR in the presence of simulated I_Kr_ blockade is not due to increased channel stochasticity, but instead reflects the intrinsic component of BVR.

When STV was plotted against average APD for individual canine ventricular myocytes or individual model cells generated based on a Gaussian distribution of conductances for all 13 currents (similar to the approach for **Figure 3A**), a non-linear relationship was obtained, which was fitted by an exponential for the purpose of parameter quantification. There was no difference in the APD dependence of STV between experiments and model, or between control and I_Kr_-block (LQT2) conditions (**Figure 8B,C**), although the fit to LQT2 simulation data had a slightly larger offset (parameter a) and lower R^2^ due to a few isolated instances with short APD but very large STV. These data indicate that the model is able to quantitatively reproduce experimental BVR characteristics covering a range of cell-to-cell differences. Moreover, these data strongly suggest that APD prolongation is the main determinant for the increased BVR in LQT2. Qualitatively similar results were obtained for I_Na_ augmentation with ATXII, although the simulated APs were very prone to EAD formation, preventing accurate STV comparison with experimental results.

**Figure 9:**
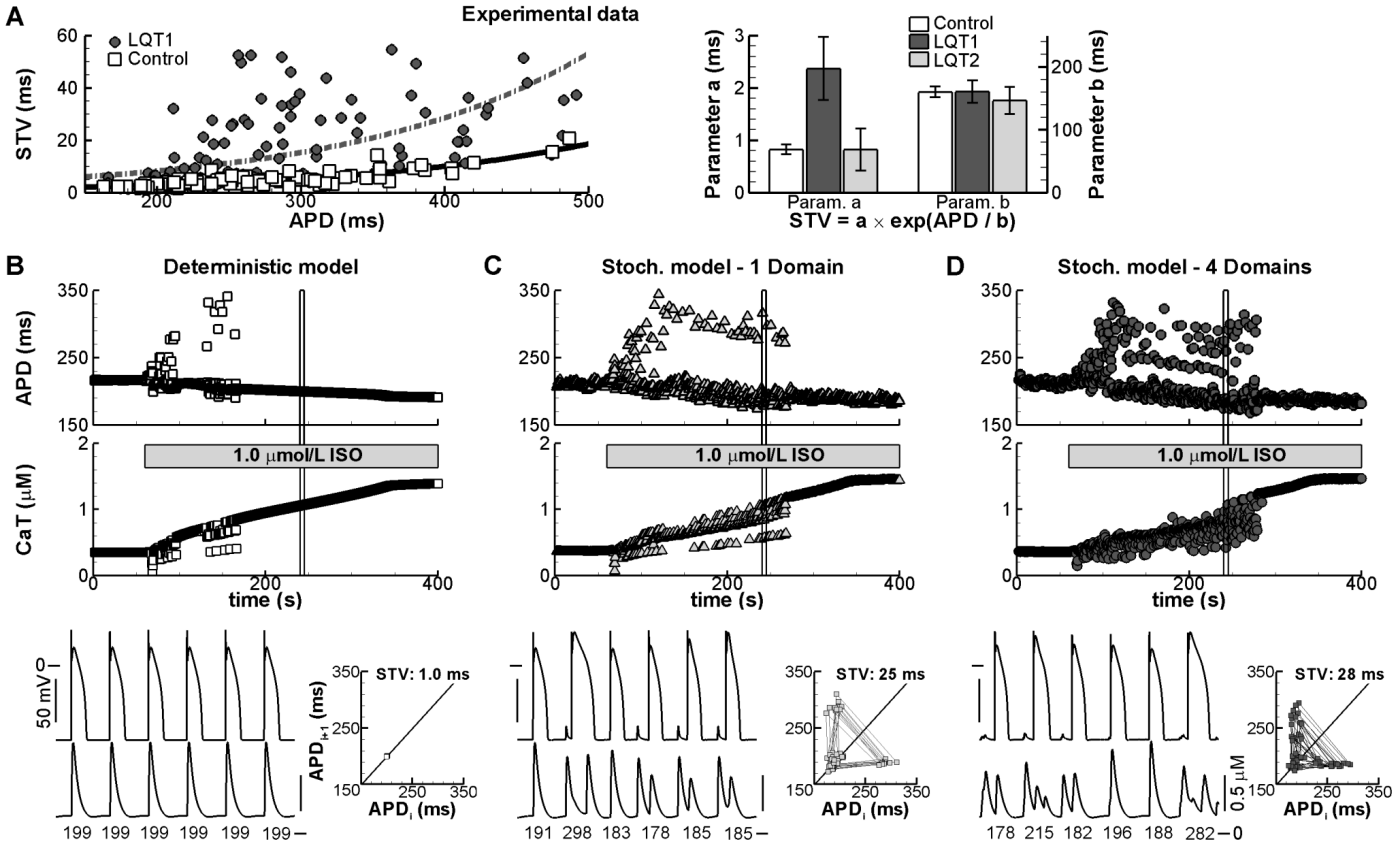
Mechanisms underlying increased BVR under LQT1 conditions with SR Ca^2+^ overload. **A.** STV vs. APD relationship under control (open symbols) or LQT1 conditions (filled symbols) in individual canine ventricular myocytes (left panel). Right panel shows the parameters of the non-linear fit of the STV vs. APD relationship under control or LQT1 conditions (solid and dashed lines in left panel, respectively), or under LQT2 conditions (from **Figure 8**). **B.** Consecutive APDs (top panel) and Ca^2+^-transient amplitudes (middle panel) during simulated application of 1.0 µmol/L isoproterenol (ISO) at a 500-ms CL in the deterministic model. Membrane potential and intracellular [Ca^2+^] for the beats indicated by the black vertical boxes are shown in the bottom panel. APD (in ms) is indicated below each beat and a Poincaré plot is shown on the right. Simulations were performed with 100% I_Ks_ inhibition to simulate LQT1 conditions and with 10% inhibition of I_NaK_, resulting in increased [Na^+^]_i_ and reduced Ca^2+^ extrusion via I_NaCa_, to promote Ca^2+^-handling abnormalities. **C.** Similar to panel B for the stochastic model with a single domain. **D.** Similar to panel B for the stochastic model divided into four identical domains connected via Ca^2+^-diffusion terms with time constant τ = 20 ms.

We have recently shown that spontaneous Ca^2+^ release (SCR) from the sarcoplasmic reticulum (SR) causes prolongation of the next APD in a pharmacological model of LQT1 (βARS and I_Ks_ inhibition), contributing to increased BVR if SCR occurs irregularly [31]. This suggests that BVR is controlled by factors other than APD alone under these conditions. In agreement, we found that experimentally recorded BVR values were substantially larger for LQT1 than for control (**Figure 9A**, left panel) or LQT2 (not shown) conditions for any given APD. The BVR vs. APD relationship showed a correspondingly larger value for parameter “a” in LQT1 (**Figure 9A**, right panel).

In the presence of 100% I_Ks_ inhibition, 10% I_NaK_ inhibition, and simulated β-adrenergic stimulation, we observed a brief period of instability in Ca^2+^ handling in the deterministic model, resulting in APD variability even in the absence of stochastic gating (**Figure 9B**). Stochastic gating significantly prolonged the window of Ca^2+^-handling abnormalities and caused pronounced APD variability during this period, in agreement with our recent experimental observations [31]. However, in the single-domain model, SCRs and the resulting delayed afterdepolarizations (DADs) had almost identical amplitudes (**Figure 9C**), resulting in two clusters of APD values, depending on the timing of the SCR. If an SCR closely preceded a beat, APD was prolonged (**Figure 9C**, bottom panel, second beat), whereas with a longer delay between the SCR and subsequent AP, APD was comparatively short. This resulted in a triangular Poincaré plot, which is not seen experimentally [8], [31].

Since it is well-established that SCR is a highly localized subcellular process [21], we hypothesized that local fluctuations in intracellular [Ca^2+^] could modulate BVR. To study the effect of subcellular variations in Ca^2+^ handling, we divided the cell into four identical domains connected via Ca^2+^ diffusion. The resulting model still falls in the category of “common-pool” models and does not reflect the dyadic nanostructure of “local-control” models. Nonetheless, the presence of local Ca^2+^ domains resulted in a wider range of SCR amplitudes and, consequently, a wider distribution of APD values compared with the stochastic model with a single domain (**Figure 9D**). These data suggest that although stochastic gating of Ca^2+^-handling proteins does not contribute to baseline BVR, it plays a critical role under conditions with SR Ca^2+^ overload. Moreover, we provide a first indication that local domains may amplify stochastic fluctuations and contribute to APD variability.

## Discussion

In this study, we developed a novel model of the canine ventricular myocyte electrophysiology including stochastic gating of all major ion currents and SR Ca^2+^-handling processes. The model showed rate dependence of APD and BVR consistent with experimental data from canine ventricular myocytes. Using this model, we obtained the following novel insights into the ionic contributors to BVR: i) stochastic channel gating (mainly of I_Na_ and I_Kr_) strongly contributes to baseline BVR; ii) BVR is more pronounced in cells with a well-developed AP plateau than in cells with triangular AP morphology; iii) BVR is reduced by cell-to-cell coupling, particularly in the case when one of the two cells has an increased BVR; iv) the rate dependence of BVR is due to “active components” and “intrinsic components”, but is independent of variations in DI; and v) APD prolongation strongly increases BVR but is not the sole determinant of exaggerated BVR in drug-induced conditions.

### Relation to existing computational models

Despite the experimental evidence of an important role for BVR as an indicator of proarrhythmic risk [2], [32], few computational models have incorporated this to date. Wilders and Jongsma were among the first to examine stochastic channel gating in a computational cardiac cell model for their investigation of beating-rate variability in sinoatrial node cells [33]. Subsequently, Tanskanen et al. employed a local control model of the canine ventricular myocyte to investigate the role of stochastic gating of I_CaL_ channels in EAD formation [13]. These authors also provided a mathematical analysis indicating that increased voltage noise skewed the distribution of APD towards longer APDs, enhancing the susceptibility to EADs [34]. In contrast, Sato et al. have shown that the EADs observed in their model of the H_2_O_2_-treated rabbit ventricular myocyte were chaotic and not due to stochastic fluctuations. However, stochastic channel gating resulted in an increased variety of temporal dynamics of the chaotic model [14]. Pueyo et al. also found that stochastic channel gating favored the occurrence of alternans and EAD generation during I_Kr_ blockade [17]. However, both Sato et al. and Pueyo et al. only considered stochastic gating of I_Ks_. Lemay et al. adapted the Luo-Rudy dynamic model of the guinea-pig ventricular myocyte to investigate the role of stochastic gating and protein turnover of a selected number of currents on APD variability and intercellular conduction delays under physiological conditions [16].

The results presented here provide a significant extension of the previously developed models by considering both stochastic gating of all major ion currents (except background currents) and Ca^2+^-handling processes. Moreover, we show that the stochastic model is quantitatively consistent with experimental measures of BVR in isolated canine ventricular myocytes and identify contributors to BVR in physiological and pathological conditions.

### BVR as a proarrhythmic marker

BVR has been proposed as a more reliable proarrhythmic marker than prolongation of repolarization *per se*, at least for specific pathological conditions [2]–[4]. Our data indicate that BVR, determined largely by stochastic channel gating during baseline conditions, is modulated by a number of factors that may play a role in arrhythmogenesis.

We find that AP morphology (**Figure 4**) and duration (**Figure 6**) affect BVR. In particular, we show that increased APD and a prolonged AP plateau increase BVR, whereas triangulation of the AP reduces BVR. As we showed in previous work [30], non-linearity of the relationship between repolarization rate and APD is, *per se*, sufficient to account for a larger impact of current fluctuations occurring during phases with very slow repolarization (plateau) on APD. Nevertheless, a prolonged AP plateau may also increase the likelihood of autoregenerative reactivation of “window” currents [35], which would boost current fluctuations. This may ultimately result in EADs, a cause of extreme temporal and spatial variability of repolarization and of ectopic impulse formation. Both Pueyo et al. [17] and Tanskanen et al. [13] have shown that stochastic fluctuations in channel gating (of I_Ks_ and I_CaL_, respectively) can indeed facilitate the development of EADs (however, see Sato et al. [15]). These data suggest that BVR reflects the robustness of repolarization and, when exaggerated, the tendency towards EAD development. It should be noted that Hondeghem et al. have previously associated drug-induced AP triangulation with increased instability and proarrhythmia in the Langendorff-perfused methoxamine-sensitized rabbit heart [36], indicating that other species-dependent factors such as the ion current profiles and the amplitude of the AP plateau may influence the effects of AP morphology on BVR.

In addition to EADs induced by reactivation of I_CaL_ during a prolonged AP plateau, abnormal Ca^2+^ handling has been shown to be able to induce EADs and delayed afterdepolarizations [31], [37]. As such, the consideration of both stochastic Ca^2+^ handling and ion-channel gating in the model presented here is important. Previous experimental data from our group have shown that buffering of intracellular Ca^2+^ (using BAPTA) can suppress BVR during βARS and I_Ks_ blockade in single ventricular myocytes [8]. Furthermore, in a pharmacological LQT2 model in intact rabbit hearts, abnormal Ca^2+^ handling also preceded fluctuations in membrane potential [38]. We found no direct contribution of individual Ca^2+^-handling proteins to BVR under baseline conditions (**Figure 2**). However, alterations in Ca^2+^ homeostasis can have a significant impact on BVR and are at least partially mediated by stochastic gating of SR Ca^2+^-handling proteins (**Figure 9**). Moreover, in the ventricular myocyte, the strong local positive feedback characteristics of Ca^2+^-induced Ca^2+^ release may amplify stochastic fluctuations within a subsarcolemmal microdomain and modulate BVR. Thus, BVR also reflects the stability of the intracellular Ca^2+^-handling system and Ca^2+^-sensitive currents.

Dispersion of repolarization has been shown to be arrhythmogenic in a variety of conditions [39]. Cell-to-cell coupling is able to suppress both temporal and spatial dispersion of repolarization (**Figure 5**), suggesting that BVR can indicate the degree of (un)coupling of the myocardium.

Combined, these data suggest that BVR reflects both the intrinsic temporal variability (stochastic channel gating and Ca^2+^ handling) as well as the sensitivity of the electrical system to these fluctuations. For example, we have shown that BVR is strongly modulated by APD, AP morphology, and cell-to-cell coupling. The integration of APD and these additional parameters may contribute to the value of BVR as proarrhythmic marker. Our results highlight an important role for abnormal Ca^2+^ handling in BVR, consistent with experimental recordings [31]. Future experimental and computational studies may elucidate the impact of Ca^2+^ on BVR at the subcellular level, providing a more extensive validation of local Ca^2+^ release and Ca^2+^-wave properties.

### Limitations and future directions

Stochastic formulations of all 13 targets were based on the well-validated characteristics of the deterministic model [18], [19] using the methodology employed in local control models [13], [21], [22]. This approach allows tracking of single-channel behavior; however, a formal validation of single-channel characteristics based on dwell times, open probability distributions, etc. is beyond the scope of this study. Furthermore, since single-channel recordings are often performed in non-physiological solutions, it would be unclear whether any deviations in single-channel behavior observed in the model under these conditions would affect the stochastic properties relevant for BVR.

We estimated the effective number of channels in the model based on experimentally obtained single-channel conductance. For several targets, the single-channel conductance or expression density is not well constrained. For example, to the best of our knowledge, there are no data on I_Ks_ single-channel conductance from native tissue, and experimental data from heterologous expression systems show considerable variability (section 2.5 of Text S1). We performed simulations over a range of channel densities to investigate the impact of this parameter (**Figure 2**). Because single-channel conductance has a large impact on BVR (**Figure 2**), the contribution of these targets may therefore be under- or overestimated.

The model presented here falls in the category of “common-pool” models that do not capture the detailed nanostructure of the ventricular myocyte where L-type Ca^2+^ channels on the T-tubular membrane and ryanodine receptors on the sarcoplasmic reticulum interact in a local nanodomain (dyad). Although we divided the model into a number of compartments and showed that this ‘local’ Ca^2+^ handling can modulate BVR (**Figure 9**), the present model cannot reproduce arrhythmogenic Ca^2+^ waves or other properties of “local-control” models that incorporate this dyadic structure. In contrast to experimental recordings [31], Poincaré plots in the presence of Ca^2+^-handling abnormalities showed a triangular pattern in the model, an effect that may be due to the limited number of domains in the local simulations. A number of “local-control” models investigating key properties of subcellular Ca^2+^ handling have recently been described [22], [40], [41]. Integration of these “local-control” models and the model presented here could facilitate the mechanistic analysis of the role of Ca^2+^-handling abnormalities in BVR in subsequent studies.

In addition to single-channel gating and Ca^2+^, other factors may modulate BVR. These factors include signaling pathways, changes in cell volume and pH, stretch and electro-mechanical feedback, etc. and are beyond the scope of the current investigation. Moreover, most of these factors will change on a timescale of minutes, whereas BVR reflects the changes in repolarization duration on the order of seconds. Thus, although these factors can affect BVR, they are likely to do so via changes in repolarization duration, Ca^2+^ handling, or stochastic channel gating that have been investigated here.

Finally, although the results presented here suggest that BVR reflects a combination of potentially proarrhythmic signals at the (sub)cellular level, its role as a marker for arrhythmogenesis can only be thoroughly investigated in a large multicellular model. The complexity of the cell model makes this computationally prohibitive for the present implementation. An alternative approach to simulate stochastic channel gating with improved computational efficiency has recently been proposed [42]. Future studies could apply this technique to study the role of BVR as a proarrhythmic marker in large-scale multicellular simulations. Our cell-pair and one-dimensional-strand simulations show that cell-to-cell coupling will reduce but not eliminate BVR. Future studies could focus on the synchronization of variability during arrhythmogenesis in a multi-scale model.

### Conclusions

We present a novel stochastic model of the canine ventricular-myocyte electrophysiology showing APD and BVR rate dependences consistent with experimental data from isolated canine ventricular myocytes under physiological conditions and in pharmacological models of LQT1-3. The model provides new insights into the (sub)cellular determinants of BVR and suggests modulating roles for several processes, including APD, AP morphology, Ca^2+^ handling, and cell-to-cell coupling. In addition to providing an important framework to further our understanding of the role that BVR can play as a proarrhythmic marker, it also gives novel insights into the differential roles of ion channels in arrhythmogenesis.

## Methods

A detailed overview of the methods employed can be found in Text S1. A brief summary of the main aspects is given below.

This investigation conformed with the Guide for the Care and Use of Laboratory Animals published by the US National Institutes of Health (NIH Publication No. 85-23, revised 1996). Animal handling was in accordance with the European Directive for the Protection of Vertebrate Animals Used for Experimental and Other Scientific Purposes (86/609/EU).

Our recent model of the canine ventricular-myocyte electrophysiology with β-adrenergic stimulation [18] formed the basis for the present study. The model was extended with i) a Markov model of the I_Kr_ including block by dofetilide, based on the work by Brennan et al. [43]; ii) a Markov model of the I_Na_ and its augmentation by ATXII; and iii) a Markov model of the RyR. For the 13 major ion currents, ion transporters and Ca^2+^-handling proteins, stochastic formulations were developed. For those proteins for which modification by phosphorylation was included in the original model [18], stochastic implementations of both the phosphorylated (P) and non-phosphorylated (NP) populations were simulated. Stochastic simulations were performed using the state-vector of the deterministic model at any given cycle length as the initial state vector. At least 250 stochastic APs were simulated in each condition to determine APD and BVR characteristics. Cell-pair experiments were simulated via a finite difference approximation of the cable equation, as previously described [19]. Both cells received an external stimulus current to eliminate the effect of depolarization differences on BVR.

The model was implemented in C (model code available as Software S1), compiled with MinGW, and simulations were run on an Intel Core I7 computer with 6 GB of RAM using a piece-wise constant time-step (0.005 ms during the AP, 0.1 ms otherwise). Data were stored in binary format with a 0.5 ms resolution and were analyzed using the mathematical software Octave. The Mersenne-Twister random number generator was used for single-channel simulations with numerical approximations for multinomial distributions as indicated in the Section 2 of Text S1.

For experimental (“wet”) studies, transmembrane APs were recorded at 37°C using high-resistance (30–60 MΩ) glass microelectrodes filled with 3 mol/L KCl in midmyocardial myocytes isolated from canine left-ventricular tissue, as previously described [8].

APD was quantified at 90% repolarization. BVR was quantified as short- or long-term variability of APD (STV or LTV) using the formulas Σ(|APD_i+1_−APD_i_|)/[n_beats_×√2] or Σ(|APD_i+1_+APD_i_−2×APD_mean_|)/[n_beats_×√2], respectively, for 30 consecutive APs, as previously described [8]. Pooled data are expressed as mean ± SD unless otherwise specified.

## Supporting Information

Figure S1 **Structure and validation of I_Kr_ Markov model properties.** **A.** Model structure. **B.** Tail I–V relationship in model and canine ventricular myocytes (reference [2] in Text S1). **C.** Time constant of activation based on a single-exponential fit in model and canine ventricular myocytes (reference [2] in Text S1). **D.** Time constant of deactivation in model and canine ventricular myocytes (reference [2] in Text S1). **E.** Dose-response curve of I_Kr_ block by dofetilide (experimental data from rabbit ventricular myocytes; reference [4] in Text S1). **F.** Use-dependent block of I_Kr_ by dofetilide in AT-1 cells (reference [3] in Text S1) and model. **G.** V_m_ dependence of dofetilide concentration required for half-maximal I_Kr_ inhibition (relative to −30 mV) in model and AT-1 cells (reference [3] in Text S1).
(TIF)

Figure S2 **Structure and validation of I_Na_ Markov model properties. A.** Model schematic of fast and late (persistent) I_Na_ components. **B.** Peak I–V relationship (left panel) and steady-state inactivation (right panel) in model (lines) and canine ventricular myocytes (symbols) at baseline or in the presence of β-adrenergic stimulation.
(TIF)

Software S1 **Implementation of stochastic model in C.**
(ZIP)

Table S1 **Number of channels/transporters simulated in the stochastic model.**
(DOC)

Text S1 **Supplemental methods.** Model definition and methodology for stochastic simulations.
(PDF)
