# Supplementary figures and images for: Determinants of Beat-to-Beat Variability of Repolarization Duration in the Canine Ventricular Myocyte: A Computational Analysis
Source: PLoS Comput Biol. 2013 Aug 22;9(8):e1003202. doi: 10.1371/journal.pcbi.1003202 (PMC3749940; doi:10.1371/journal.pcbi.1003202)

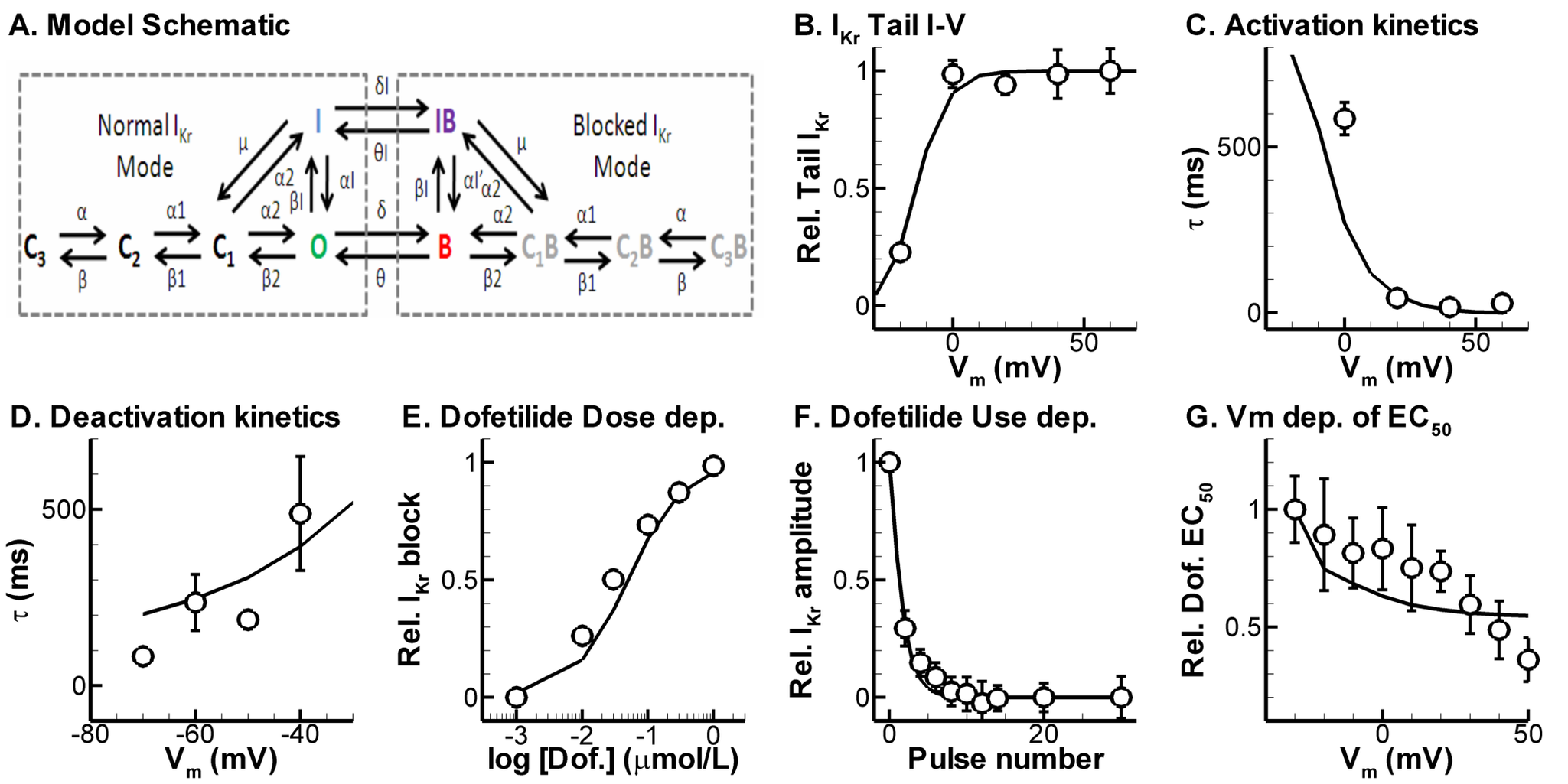

Supplement: Figure S1 — Structure and validation of IKr Markov model properties. A. Model structure. B. Tail I–V relationship in model and canine ventricular myocytes (reference [2] in Text S1). C. Time constant of activation based on a single-exponential fit in model and canine ventricular myocytes (reference [2] in Text S1). D. Time constant of deactivation in model and canine ventricular myocytes (reference [2] in Text S1). E. Dose-response curve of IKr block by dofetilide (experimental data from rabbit ventricular myocytes; reference [4] in Text S1). F. Use-dependent block of IKr by dofetilide in AT-1 cells (reference [3] in Text S1) and model. G. Vm dependence of dofetilide concentration required for half-maximal IKr inhibition (relative to −30 mV) in model and AT-1 cells (reference [3] in Text S1). (TIF) [file pcbi.1003202.s001.tif]

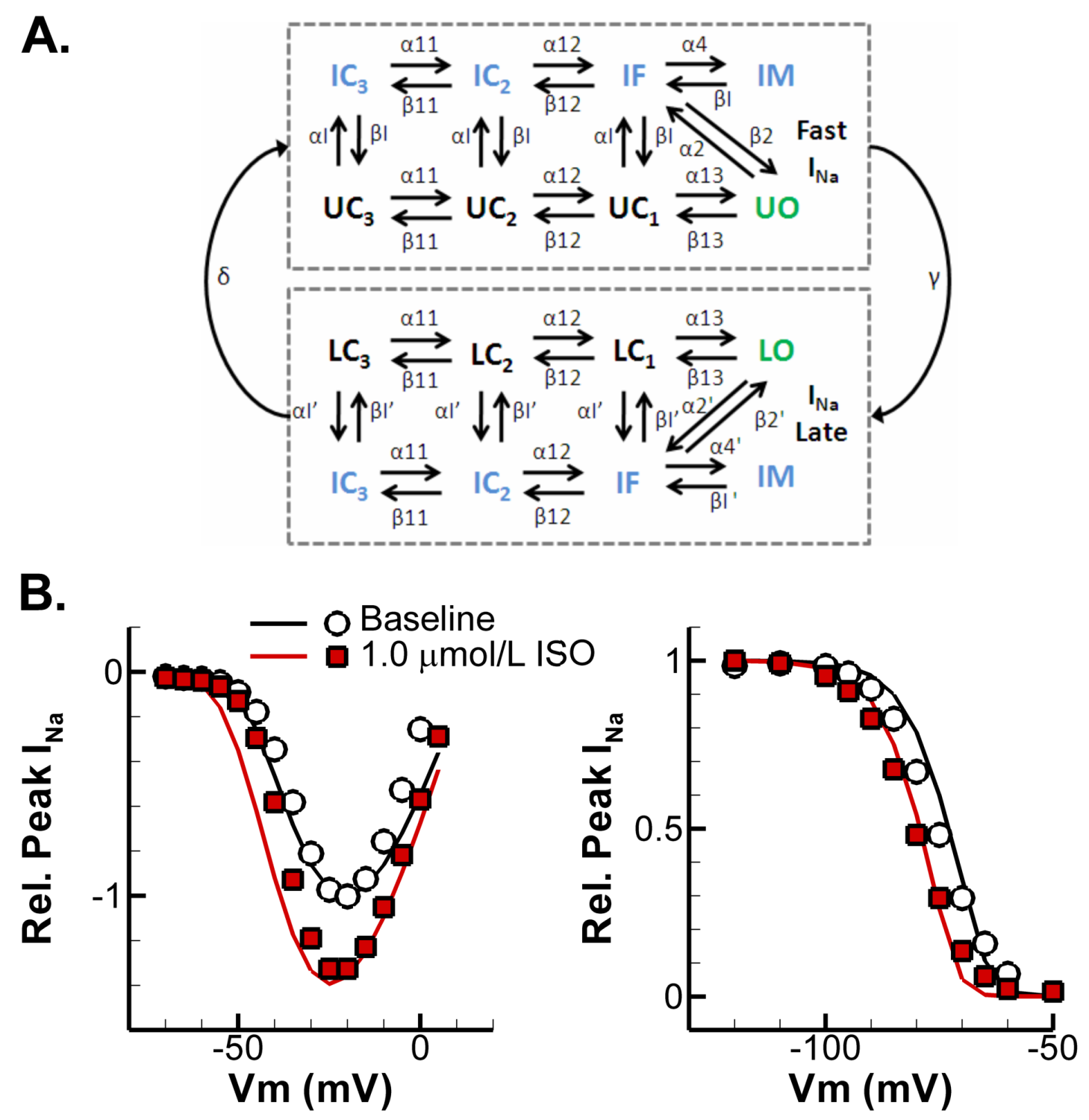

Supplement: Figure S2 — Structure and validation of INa Markov model properties. A. Model schematic of fast and late (persistent) INa components. B. Peak I–V relationship (left panel) and steady-state inactivation (right panel) in model (lines) and canine ventricular myocytes (symbols) at baseline or in the presence of β-adrenergic stimulation. (TIF) [file pcbi.1003202.s002.tif]
