# Supplementary material for: Determinants of Beat-to-Beat Variability of Repolarization Duration in the Canine Ventricular Myocyte: A Computational Analysis
Source: PLoS Comput Biol. 2013 Aug 22;9(8):e1003202. doi: 10.1371/journal.pcbi.1003202 (PMC3749940; doi:10.1371/journal.pcbi.1003202)
Supplement: Table S1 — Number of channels/transporters simulated in the stochastic model. (DOC) [file pcbi.1003202.s004.doc]

| **Current** | **# Channels** | **Reference** |
| --- | --- | --- |
| **I_CaL_** | 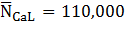 | Based on a single-channel conductance of 3.0 pS (at 2.0 mmol/L extracellular Ca^2+^ in rat ventricular myocytes (references listed in Text S1). Consistent with ~80,000 channels as in Restrepo et al. . |
| **I_Cl(Ca)_** | 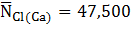 | Based on a single-channel conductance of 0.91 pS (at 150 mmol/L extracellular Cl^-^ and 5.0 mmol/L intracellular Cl^-^ in canine ventricular myocytes . |
| **I_K1_** | 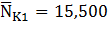 | A single-channel conductance of approximately 30 pS has been reported at high (150 mmol/L) [K^+^]_o_ in a number of species, including canine ventricular myocytes . Based on a square-root dependence of channel conductance on [K^+^]_o_, a single-channel conductance of approximately 5.0 pS is obtained at physiological [K^+^]_o_. |
| **I_Kr_** | 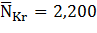 | Using a single-channel conductance of 2.25 pS as observed in mouse and rabbit ventricular myocytes, after a similar correction as applied for I_K1_. |
| **I_Ks_** | 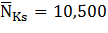 | Based on a single-channel conductance of 4.50 pS obtained in Xenopus oocytes expressing KCNQ1 and KCNE1 . There are, to the best of our knowledge, no data available from cardiac myocytes. Estimates in heterologous expression systems range from 0.58 pS to 16 pS . |
| **I_Kur_** | 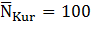 | Yue et al. determined a single-channel conductance of 20.3 pS in canine atrial myocytes at 5.4 mmol/L [K^+^]_o_ . The low number of channels is consistent with the presence, but very low expression of an I_Kur_-like channel in canine ventricle . |
| **I_Na_** | 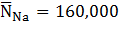 | Using a single-channel conductance of 20.1 pS as observed in canine, rabbit and guinea-pig ventricular myocytes . |
| **I_NaCa_** | 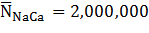 | Based on a density of 200-400 exchangers per μm^2^ . |
| **I_NaK_** | 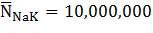 | Pump-density estimates based on ouabain-sensitive transient currents range between 1000 and 3200 exchangers per μm^2^ in ventricular myocytes . We employ the ratio of approximately 1 NaCa : 5 NaK that follows from these density estimates. |
| **I_pCa_** | 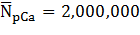 | Assumed to be similar to 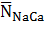. |
| **I_To_** | 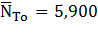 | Based on a single-channel conductance of 13.0 pS as measured by Fedida and Giles in rabbit ventricular myocytes . |
| **I_rel_** | 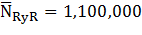 | RyR density is 10 fold larger than 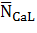, consistent with previously published local control models . |
| **I_up_** | 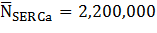 | Assumed to be of similar magnitude as 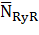. |
