# Supplementary material for: Determinants of Beat-to-Beat Variability of Repolarization Duration in the Canine Ventricular Myocyte: A Computational Analysis
Source: PLoS Comput Biol. 2013 Aug 22;9(8):e1003202. doi: 10.1371/journal.pcbi.1003202 (PMC3749940; doi:10.1371/journal.pcbi.1003202)
Supplement: Text S1 — Supplemental methods. Model definition and methodology for stochastic simulations. (PDF) [file pcbi.1003202.s005.pdf]

Determinants of Beat-to-Beat Variability of Repolarization Duration in the Canine Ventricular Myocyte: a Computational Analysis

Online Data Supplement

Jordi Heijman, Antonio Zaza, Daniel M. Johnson, Yoram Rudy, Ralf L.M. Peeters, Paul G.A. Volders* and Ronald L. Westra*

* These authors contributed equally to this work

Corresponding Authors:

Ronald L. Westra, PhD
Dept. of Knowledge Engineering
Faculty of Humanities and Sciences
Maastricht University
P.O. Box 616
6200 MD Maastricht
The Netherlands
e-mail: westra@maastrichtuniversity.nl
phone: +31 43 3882270
fax: + 31 43 3884910

Paul G.A. Volders, MD, PhD
Dept. of Cardiology
Cardiovascular Research Institute Maastricht
Maastricht University Medical Centre
P.O. Box 5800
6202 AZ Maastricht
The Netherlands
e-mail: p.volders@maastrichtuniversity.nl
phone: +31 43 3875094
fax: +31 43 3875104

Extended Methods

1. Adaptations to the HRdβAR model

1.1 Markov model formulation of the rapidly-activating delayed-rectifier K^+ current (I_{Kr})

The original Hodgkin-Huxley based formulation of I_{Kr} was replaced with a 10-state Markov model based on the model structure described by Silva and Rudy [1] extended with state-dependent block of I_{Kr} channels by dofetilide (**Figure S1A**). Model parameters were adjusted to reproduce I_{Kr} peak and tail I-V relationships, time constants of activation and time constants of deactivation measured in canine ventricular myocytes [2] (**Figure S1B-D**). Channels can enter the blocked mode via open and inactivated states, based on a V_m-dependent modulation of the dofetilide EC_{50} [3].The model reproduces dofetilide dose-response relationship measured in rabbit ventricular myocytes [4], use-dependence of relative inhibition as determined in AT-1 cells, [3] and V_m-dependence of dofetilide EC_{50} [3] (**Figure S1E-G**).

Equations for the updated I_{Kr} formulation are as follows:

$$\alpha_{Kr,2} = 4.8712 \cdot 10^{-3} \cdot \exp\left(2.5082 \cdot V_m \cdot \frac{F}{R \cdot T}\right)$$

$$\beta_{Kr,2} = 5.0582 \cdot 10^{-4} \cdot \exp\left(0.8035 \cdot V_m \cdot \frac{F}{R \cdot T}\right)$$

$$\alpha_{Kr,1} = 3.1224, \beta_{Kr,1} = 2.3685$$

$$\alpha_{Kr} = 1.1070 \cdot 10^{-2} \cdot \exp\left(1.5816 \cdot V_m \cdot \frac{F}{R \cdot T}\right)$$

$$\beta_{Kr} = 3.7892 \cdot 10^{-3} \cdot \exp\left(1.1872 \cdot V_m \cdot \frac{F}{R \cdot T}\right)$$

$$\alpha_{Kr,i} = 4.8457 \cdot 10^{-1} \cdot \exp\left(0.7296 \cdot V_m \cdot \frac{F}{R \cdot T}\right) \cdot \frac{4.5}{[K^+]_o}$$

$$\beta_{Kr,i} = 1.2120 \cdot \exp\left(0.5816 \cdot V_m \cdot \frac{F}{R \cdot T}\right) \cdot \left(\frac{4.5}{[K^+]_o}\right)^{0.3}$$

$$\mu_{Kr} = \frac{\alpha_{Kr,i} \cdot \beta_{Kr,2}}{\beta_{Kr,i}}$$

$k_{Dof,on} = 5.2353 \cdot 10^{-3} \cdot [Dof]$, $k_{Dof,off} = 3.2172 \cdot 10^{-4}$

$k_{Dof,on,i} = 2.7898 \cdot 10^{-2} \cdot [Dof]$, $k_{Dof,off,i} = 8.7055 \cdot 10^{-4}$

$$\alpha_{Kr,i,Dof} = \frac{k_{Dof,off,i} \cdot \alpha_{Kr,i} \cdot k_{Dof,on}}{k_{Dof,off} \cdot k_{Dof,on,i}}$$

$$\mu_{Kr,Dof} = \frac{\alpha_{Kr,i,Dof} \cdot \beta_{Kr,2}}{\beta_{Kr,i}}$$

$$\frac{dC3_{Kr}}{dt} = \beta_{Kr} \cdot C2_{Kr} - \alpha_{Kr} \cdot C3_{Kr}$$

$$\frac{dC2_{Kr}}{dt} = \beta_{Kr,1} \cdot C1_{Kr} + \alpha_{Kr} \cdot C3_{Kr} - \left(\alpha_{Kr,1} + \beta_{Kr}\right) \cdot C2_{Kr}$$

$$\frac{dC1_{Kr}}{dt} = \alpha_{Kr,1} \cdot C2_{Kr} + \beta_{Kr,2} \cdot O_{Kr} + \mu_{Kr} \cdot In_{Kr} - \left(2 \cdot \alpha_{Kr,2} + \beta_{Kr,1}\right) \cdot C1_{Kr}$$

$$\frac{dO_{Kr}}{dt} = \alpha_{Kr,i} \cdot In_{Kr} + \alpha_{Kr,2} \cdot C1_{Kr} + k_{Dof,off} \cdot B_{Kr} - \left(\beta_{Kr,i} + \beta_{Kr,2} + k_{Dof,on}\right) \cdot O_{Kr}$$

$$\frac{dIn}{dt} = \alpha_{Kr,2} \cdot C1_{Kr} + \beta_{Kr,i} \cdot O_{Kr} + k_{Dof,off,i} \cdot InB_{Kr} - \left(\mu_{Kr} + \alpha_{Kr,i} + k_{Dof,on,i}\right) \cdot In_{Kr}$$

$$\frac{dC3B_{Kr}}{dt} = \beta_{Kr} \cdot C2B_{Kr} - \alpha_{Kr} \cdot C3B_{Kr}$$

$$\frac{dC2B_{Kr}}{dt} = \beta_{Kr,1} \cdot C1B_{Kr} + \alpha_{Kr} \cdot C3B_{Kr} - \left(\alpha_{Kr,1} + \beta_{Kr}\right) \cdot C2B_{Kr}$$

$$\frac{dC1B_{Kr}}{dt} = \alpha_{Kr,1} \cdot C2B_{Kr} + \beta_{Kr,2} \cdot B_{Kr} + \mu_{Kr,Dof} \cdot InB_{Kr} - \left(2 \cdot \alpha_{Kr,2} + \beta_{Kr,1}\right) \cdot C1B_{Kr}$$

$$\frac{dB_{Kr}}{dt} = \alpha_{Kr,i,Dof} \cdot InB_{Kr} + \alpha_{Kr,2} \cdot C1B_{Kr} + k_{Dof,on} \cdot O_{Kr} - \left(\beta_{Kr,i} + \beta_{Kr,2} + k_{Dof,off}\right) \cdot B_{Kr}$$

$$\frac{dInB}{dt} = \alpha_{Kr,2} \cdot C1B_{Kr} + \beta_{Kr,i} \cdot B_{Kr} + k_{Dof,on,i} \cdot In_{Kr} - \left(\mu_{Kr,Dof} + \alpha_{Kr,i,Dof} + k_{Dof,off,i}\right) \cdot InB_{Kr}$$

$$G_{Kr} = 5.2530 \cdot 10^{-3} \cdot ([K^+]_o)^{0.59}$$

$$I_{Kr} = G_{Kr} \cdot O_{Kr} \cdot (V_m - E_K)$$

1.2 Markov model formulation of the Na^+ current (I_{Na})

The Markov-model structure of Clancy and Rudy [5] was employed for both normal inactivation and slow inactivation populations (**Figure S2**). The overall model structure is slightly different from the original model by Clancy and Rudy where one additional row of four states was added to simulate persistent I_{Na}. This change was necessary to reproduce characteristics of I_{Na} and I_{NaL} from the original Hodgkin-Huxley-based formulation, including PKA- and CaMKII-dependent alterations [6]. In particular, I_{NaL} in the original model has identical activation properties as I_{Na} but shows slowed (albeit noticeable) inactivation. As such, both tiers of the Markov model have inactivated states and identical activation rates. In contrast, rates of inactivation are much slower in the lower tier. Model I_{Na} peak I-V relationship and steady-state inactivation are consistent with those measured in isolated canine ventricular myocytes [7].

Equations for the updated I_{Na} formulation are as follows:

$$\alpha_{Na,11}^{NP} = \frac{4.5170}{6.6161 \cdot 10^{-2} \cdot \exp\left(-\frac{V_m}{18.033}\right) + 2.1276 \cdot 10^{-1} \cdot \exp\left(-\frac{V_m}{167.77}\right)}$$

$$\alpha_{Na,12}^{NP} = \frac{4.5170}{6.6161 \cdot 10^{-2} \cdot \exp\left(-\frac{V_m}{24.237}\right) + 1.7127 \cdot 10^{-1} \cdot \exp\left(-\frac{V_m}{167.77}\right)}$$

$$\alpha_{Na,13}^{NP} = \frac{4.5170}{6.6161 \cdot 10^{-2} \cdot \exp\left(-\frac{V_m}{13.038}\right) + 2.7731 \cdot 10^{-1} \cdot \exp\left(-\frac{V_m}{167.77}\right)}$$

$$\beta_{Na,11}^{NP} = 1.7687 \cdot 10^{-1} \cdot \exp\left(-\frac{V_m}{15.905}\right)$$

$$\beta_{Na,12}^{NP} = 2.2240 \cdot 10^{-1} \cdot \exp\left(-\frac{V_m - 6.1967}{15.905}\right)$$

$$\beta_{Na,13}^{NP} = 2.9500 \cdot 10^{-1} \cdot \exp\left(-\frac{V_m - 10.891}{15.905}\right)$$

$$\alpha_{Na,2}^{NP} = 6.3736 \cdot \exp\left(\frac{V_m + 3.3271 \cdot f_{INa}^{P,CaMKII}}{109.64}\right), \qquad \alpha_{Na,2l}^{NP} = \alpha_{Na,2}^{NP} \cdot 1.8366 \cdot 10^{-4}$$

$$\beta_{Na,i}^{NP} = 8.0991 \cdot 10^{-8} \cdot \exp\left(-\frac{V_m + 3.3271 \cdot f_{INa}^{P,CaMKII}}{5.6611}\right), \qquad \beta_{Na,il}^{NP} = \beta_{Na,i}^{NP} \cdot 1.1014 \cdot 10^{-2}$$

$$\alpha_{Na,4}^{NP} = \frac{\beta_{Na,i}^{NP}}{9.2183}, \qquad \alpha_{Na,4l}^{NP} = \alpha_{Na,4}^{NP} \cdot 1.1014 \cdot 10^{-2}$$

$$\alpha_{Na,i}^{NP} = 3.1367 \cdot 10^{-2} + 1.6356 \cdot 10^{-5} \cdot \left(V_m + 3.3271 \cdot f_{INa}^{P,CaMKII}\right), \qquad \alpha_{Na,il}^{NP} = \alpha_{Na,i}^{NP} \cdot 1.1014 \cdot 10^{-2}$$

$$\beta_{Na,2}^{NP} = \frac{\alpha_{Na,13}^{NP} \cdot \alpha_{Na,2}^{NP} \cdot \beta_{Na,i}^{NP}}{\beta_{Na,13}^{NP} \cdot \alpha_{Na,i}^{NP}}, \qquad \beta_{Na,2l}^{NP} = \frac{\alpha_{Na,13}^{NP} \cdot \alpha_{Na,2l}^{NP} \cdot \beta_{Na,il}^{NP}}{\beta_{Na,13}^{NP} \cdot \alpha_{Na,il}^{NP}}$$

$$\gamma_{Na}^{NP} = 1.3053 \cdot 10^{-7} + 2.4015 \cdot 10^{-7} \cdot f_{INa}^{P,CaMKII} + \frac{2.90 \cdot 10^{-7}}{1 + \left(\frac{4.0}{[ATXII]}\right)^2}$$

$$\delta_{Na}^{NP} = 4.8365 \cdot 10^{-4}$$

$$f_{Na,slow}^{NP} = \frac{\gamma_{Na}^{NP}}{\gamma_{Na}^{NP} + \delta_{Na}^{NP}}$$

$$\alpha_{Na,11}^{P} = \frac{8.3241}{6.6161 \cdot 10^{-2} \cdot \exp\left(-\frac{V_m}{18.033}\right) + 2.1276 \cdot 10^{-1} \cdot \exp\left(-\frac{V_m}{167.77}\right)}$$

$$\alpha_{Na,12}^{P} = \frac{8.3241}{6.6161 \cdot 10^{-2} \cdot \exp\left(-\frac{V_m}{24.237}\right) + 1.7127 \cdot 10^{-1} \cdot \exp\left(-\frac{V_m}{167.77}\right)}$$

$$\alpha_{Na,13}^{P} = \frac{8.3241}{6.6161 \cdot 10^{-2} \cdot \exp\left(-\frac{V_m}{13.038}\right) + 2.7731 \cdot 10^{-1} \cdot \exp\left(-\frac{V_m}{167.77}\right)}$$

$$\beta_{Na,11}^{P} = 1.0336 \cdot 10^{-1} \cdot \exp\left(-\frac{V_m}{15.905}\right)$$

$$\beta_{Na,12}^{P} = 2.2972 \cdot 10^{-1} \cdot \exp\left(-\frac{V_m - 6.1967}{15.905}\right)$$

$$\beta_{Na,13}^{P} = 2.6387 \cdot 10^{-1} \cdot \exp\left(-\frac{V_m - 10.891}{15.905}\right)$$

$$\alpha_{Na,2}^{P} = 5.3095 \cdot \exp\left(\frac{V_m + 0.5625 \cdot f_{INa}^{P,CaMKII}}{109.64}\right), \qquad \alpha_{Na,2l}^{P} = \alpha_{Na,2}^{P} \cdot 1.4827 \cdot 10^{-4}$$

$$\beta_{Na,i}^{P} = 2.9162 \cdot 10^{-8} \cdot \exp\left(-\frac{V_m + 0.5625 \cdot f_{INa}^{P,CaMKII}}{5.6779}\right), \qquad \beta_{Na,il}^{NP} = \beta_{Na,i}^{NP} \cdot 2.6707 \cdot 10^{-2}$$

$$\alpha_{Na,4}^{P} = \frac{\beta_{Na,i}^{P}}{6.0179}, \qquad \alpha_{Na,4l}^{P} = \alpha_{Na,4}^{P} \cdot 2.6707 \cdot 10^{-2}$$

$$\alpha_{Na,i}^{P} = 3.1367 \cdot 10^{-2} + 7.9063 \cdot 10^{-6} \cdot \left(V_m + 0.5625 \cdot f_{INa}^{P,CaMKII}\right), \qquad \alpha_{Na,il}^{P} = \alpha_{Na,i}^{P} \cdot 2.6707 \cdot 10^{-2}$$

$$\beta^{P}_{Na,2} = \frac{\alpha^{P}_{Na,13} \cdot \alpha^{P}_{Na,2} \cdot \beta^{P}_{Na,i}}{\beta^{P}_{Na,13} \cdot \alpha^{P}_{Na,i}}, \qquad \beta^{NP}_{Na,2l} = \frac{\alpha^{P}_{Na,13} \cdot \alpha^{P}_{Na,2l} \cdot \beta^{P}_{Na,il}}{\beta^{P}_{Na,13} \cdot \alpha^{P}_{Na,il}}$$

$$\gamma^{P}_{Na} = 1.4099 \cdot 10^{-7} + 2.1616 \cdot 10^{-7} \cdot f^{P,CaMKII}_{INa} + \frac{2.90 \cdot 10^{-7}}{1 + \left(\frac{4.0}{[ATXII]}\right)^2}$$

$$\delta^{P}_{Na} = 4.6975 \cdot 10^{-4}$$

$$f^{P}_{Na,slow} = \frac{\gamma^{P}_{Na}}{\gamma^{P}_{Na} + \delta^{P}_{Na}}$$

For $x \in \{NP, P\}$:

$$\frac{dLC3^{x}_{Na}}{dt} = \beta^{x}_{Na,11} \cdot LC2^{x}_{Na} + \beta^{x}_{Na,il} \cdot LIC3^{x}_{Na} - \left(\alpha^{x}_{Na,11} + \alpha^{x}_{Na,il}\right) \cdot LC3^{x}_{Na}$$

$$\frac{dLC2^{x}_{Na}}{dt} = \alpha^{x}_{Na,11} \cdot LC3^{x}_{Na} + \beta^{x}_{Na,12} \cdot LC1^{x}_{Na} + \beta^{x}_{Na,il} \cdot LIC2^{x}_{Na} - \left(\alpha^{x}_{Na,12} + \beta^{x}_{Na,11} + \alpha^{x}_{Na,il}\right) \cdot LC2^{x}_{Na}$$

$$\frac{dLC1^{x}_{Na}}{dt} = \alpha^{x}_{Na,12} \cdot LC2^{x}_{Na} + \beta^{x}_{Na,13} \cdot LO^{x}_{Na} + \beta^{x}_{Na,il} \cdot LIC1^{x}_{Na} - \left(\alpha^{x}_{Na,13} + \beta^{x}_{Na,12} + \alpha^{x}_{Na,il}\right) \cdot LC1^{x}_{Na}$$

$$\frac{dLO^{x}_{Na}}{dt} = \alpha^{x}_{Na,13} \cdot LC1^{x}_{Na} + \beta^{x}_{Na,2l} \cdot LIF^{x}_{Na} - \left(\beta^{x}_{Na,13} + \alpha^{x}_{Na,2l}\right) \cdot LO^{x}_{Na}$$

$$\frac{dUC3^{x}_{Na}}{dt} = \beta^{x}_{Na,11} \cdot LC2^{x}_{Na} + \beta^{x}_{Na,i} \cdot LIC3^{x}_{Na} - \left(\alpha^{x}_{Na,11} + \alpha^{x}_{Na,i}\right) \cdot UC3^{x}_{Na}$$

$$\frac{dUC2^{x}_{Na}}{dt} = \alpha^{x}_{Na,11} \cdot LC3^{x}_{Na} + \beta^{x}_{Na,12} \cdot LC1^{x}_{Na} + \beta^{x}_{Na,i} \cdot LIC2^{x}_{Na} - \left(\alpha^{x}_{Na,12} + \beta^{x}_{Na,11} + \alpha^{x}_{Na,i}\right) \cdot UC2^{x}_{Na}$$

$$\frac{dUC1^{x}_{Na}}{dt} = \alpha^{x}_{Na,12} \cdot LC2^{x}_{Na} + \beta^{x}_{Na,13} \cdot LO^{x}_{Na} + \beta^{x}_{Na,i} \cdot LIF^{x}_{Na} - \left(\alpha^{x}_{Na,13} + \beta^{x}_{Na,12} + \alpha^{x}_{Na,i}\right) \cdot UC1^{x}_{Na}$$

$$\frac{dUO^{x}_{Na}}{dt} = \alpha^{x}_{Na,13} \cdot LC1^{x}_{Na} + \beta^{x}_{Na,2} \cdot LIF^{x}_{Na} - \left(\beta^{x}_{Na,13} + \alpha^{x}_{Na,2}\right) \cdot UO^{x}_{Na}$$

$$\frac{dLIC3^{x}_{Na}}{dt} = \beta^{x}_{Na,11} \cdot LIC2^{x}_{Na} + \alpha^{x}_{Na,il} \cdot LC3^{x}_{Na} - \left(\alpha^{x}_{Na,11} + \beta^{x}_{Na,il}\right) \cdot LIC3^{x}_{Na}$$

$$\frac{dLIC2^{x}_{Na}}{dt} = \alpha^{x}_{Na,11} \cdot LIC3^{x}_{Na} + \beta^{x}_{Na,12} \cdot LIF^{x}_{Na} + \alpha^{x}_{Na,il} \cdot LC2^{x}_{Na} - \left(\alpha^{x}_{Na,12} + \beta^{x}_{Na,11} + \beta^{x}_{Na,il}\right) \cdot LIC2^{x}_{Na}$$

$$\frac{dLIF_{Na}^{x}}{dt} = \alpha_{Na,12}^{x} \cdot LIC2_{Na}^{x} + \beta_{Na,il}^{x} \cdot LIM_{Na}^{x} + \alpha_{Na,il}^{x} \cdot LC1_{Na}^{x} + \alpha_{Na,2}^{x} \cdot LO_{Na}^{x} - \left(\alpha_{Na,4l}^{x} + \beta_{Na,12}^{x} + \beta_{Na,2l}^{x} + \beta_{Na,il}^{x}\right) \cdot LIF_{Na}^{x}$$

$$\frac{dLIM_{Na}^{x}}{dt} = \alpha_{Na,4l}^{x} \cdot LIF_{Na}^{x} - \beta_{Na,il}^{x} \cdot LIM_{Na}^{x}$$

$$\frac{dUIC3_{Na}^{x}}{dt} = \beta_{Na,11}^{x} \cdot UIC2_{Na}^{x} + \alpha_{Na,i}^{x} \cdot UC3_{Na}^{x} - \left(\alpha_{Na,11}^{x} + \beta_{Na,i}^{x}\right) \cdot UIC3_{Na}^{x}$$

$$\frac{dUIC2_{Na}^{x}}{dt} = \alpha_{Na,11}^{x} \cdot UIC3_{Na}^{x} + \beta_{Na,12}^{x} \cdot UIF_{Na}^{x} + \alpha_{Na,i}^{x} \cdot UC2_{Na}^{x} - \left(\alpha_{Na,12}^{x} + \beta_{Na,11}^{x} + \beta_{Na,i}^{x}\right) \cdot UIC2_{Na}^{x}$$

$$\frac{dUIF_{Na}^{x}}{dt} = \alpha_{Na,12}^{x} \cdot UIC2_{Na}^{x} + \beta_{Na,i}^{x} \cdot UIM_{Na}^{x} + \alpha_{Na,i}^{x} \cdot UC1_{Na}^{x} + \alpha_{Na,2}^{x} \cdot UO_{Na}^{x} - \left(\alpha_{Na,4}^{x} + \beta_{Na,12}^{x} + \beta_{Na,2}^{x} + \beta_{Na,i}^{x}\right) \cdot UIF_{Na}^{x}$$

$$\frac{dUIM_{Na}^{x}}{dt} = \alpha_{Na,4}^{x} \cdot UIF_{Na}^{x} - \beta_{Na,i}^{x} \cdot UIM_{Na}^{x}$$

$$I_{Na}^{NP} = 11.188 \cdot \left(\left(1 - f_{Na,slow}^{NP}\right) \cdot UO_{Na}^{NP} + f_{Na,slow}^{NP} \cdot LO_{Na}^{NP}\right) \cdot (V_m - E_{Na})$$

$$I_{Na}^{P} = 11.188 \cdot \left(\left(1 - f_{Na,slow}^{P}\right) \cdot UO_{Na}^{P} + f_{Na,slow}^{P} \cdot LO_{Na}^{P}\right) \cdot (V_m - E_{Na})$$

$$I_{Na} = \left(1 - \hat{f}_{INa}^{PKA}\right) \cdot I_{Na}^{NP} + \hat{f}_{INa}^{PKA} \cdot I_{Na}^{P}$$

1.3 Markov model formulation of sarcoplasmic reticulum (SR) Ca^{2+} release

Ca^{2+}-induced Ca^{2+} release (CICR) in the original HRdβAR model depends directly on the L-type Ca^{2+} current (I_{CaL}) based on the formulation by Livshitz et al. [8]. It is difficult to create a stochastic version of this formulation since it does not exhibit the strong positive feedback characteristic for CICR (where the Ca^{2+} released by some ryanodine receptors (RyRs) may activate other RyRs in the same microdomain), which likely affects its stochastic properties. As such, the original phosphorylated and non-phosphorylated CICR formulations were replaced by two 4-state Markov Models proposed by Restrepo et al. in their local control model of Ca^{2+} handling in the rabbit ventricular myocyte [9]. Model parameters were adjusted to reproduce rate-dependent Ca^{2+} transient (CaT) properties (amplitude, time to peak, and time constant of

decay) of the original HRdβAR model in the absence or presence of β-adrenergic stimulation (βARS).

Equations for the updated release formulation are as follows:

$$\widehat{M}_{RyR}^{NP} = \frac{1}{1 + \left(\frac{[Ca^{2+}]_{JSR}}{0.4163}\right)^{15.24}}$$

$$Ac_{RyR,\infty}^{NP} = 3.9665 \cdot 10^{-5} \cdot \left(\exp\left(\frac{[Ca^{2+}]_{JSR}}{2.4898}\right) - 1\right) + \frac{0.032}{1 + \left(\frac{1.1960 \cdot 10^{-3} \cdot \left(1 + 2.0 \cdot f_{RYR}^{P,CaMKII}\right)}{[Ca^{2+}]_{ss,CaL}}\right)^{8}}$$

$$Acl_{RyR,\infty}^{NP} = 3.9665 \cdot 10^{-5} \cdot \left(\exp\left(\frac{[Ca^{2+}]_{JSR}}{2.4898}\right) - 1\right) + \frac{0.0005}{1 + \left(\frac{0.003 \cdot \left(1 + 2.0 \cdot f_{RYR}^{P,CaMKII}\right)}{[Ca^{2+}]_{ss,CaL}}\right)^{8}}$$

$$\tau_{RyR,Ac}^{NP} = 0.2 + 0.5000 \cdot \widehat{M}_{RyR}^{NP}$$

$$k_{RyR,12}^{NP} = \frac{Ac_{RyR,\infty}^{NP}}{\tau_{RyR,Ac}^{NP}}, \qquad k_{RyR,21}^{NP} = \frac{1 - Ac_{RyR,\infty}^{NP}}{\tau_{RyR,Ac}^{NP}}$$

$$k_{RyR,43}^{NP} = \frac{Acl_{RyR,\infty}^{NP}}{\tau_{RyR,Ac}^{NP}}, \qquad k_{RyR,34}^{NP} = \frac{1 - Acl_{RyR,\infty}^{NP}}{\tau_{RyR,Ac}^{NP}}$$

$$k_{RyR,14,23}^{NP} = \frac{\widehat{M}_{RyR}^{NP}}{0.5250}, \qquad k_{RyR,41}^{NP} = \frac{1}{395.19}$$

$$k_{RyR,32}^{NP} = \frac{k_{RyR,41}^{NP} \cdot k_{RyR,12}^{NP} \cdot k_{RyR,34}^{NP}}{k_{RyR,43}^{NP} \cdot k_{RyR,21}^{NP}}$$

$$\frac{dCB_{RyR}^{NP}}{dt} = k_{RyR,21}^{NP} \cdot OB_{RyR}^{NP} + k_{RyR,41}^{NP} \cdot CU_{RyR}^{NP} - \left(k_{RyR,12}^{NP} + k_{RyR,14,23}^{NP}\right) \cdot CB_{RyR}^{NP}$$

$$\frac{dOB_{RyR}^{NP}}{dt} = k_{RyR,12}^{NP} \cdot CB_{RyR}^{NP} + k_{RyR,32}^{NP} \cdot OU_{RyR}^{NP} - \left(k_{RyR,21}^{NP} + k_{RyR,14,23}^{NP}\right) \cdot OB_{RyR}^{NP}$$

$$\frac{dOU_{RyR}^{NP}}{dt} = k_{RyR,14,23}^{NP} \cdot OB_{RyR}^{NP} + k_{RyR,43}^{NP} \cdot CU_{RyR}^{NP} - \left(k_{RyR,32}^{NP} + k_{RyR,34}^{NP}\right) \cdot OU_{RyR}^{NP}$$

$$\frac{dCU_{RyR}^{NP}}{dt} = k_{RyR,14,23}^{NP} \cdot CB_{RyR}^{NP} + k_{RyR,34}^{NP} \cdot OU_{RyR}^{NP} - \left(k_{RyR,41}^{NP} + k_{RyR,43}^{NP}\right) \cdot CU_{RyR}^{NP}$$

$$I_{Rel}^{NP} = \frac{3.5289 \cdot 10^{-5}}{V_{ss}} \cdot \left(OB_{RyR}^{NP} + OU_{RyR}^{NP}\right) \cdot \left([Ca^{2+}]_{JSR} - [Ca^{2+}]_{ss,CaL}\right)$$

$$\widehat{M}_{RyR}^{P} = \frac{1}{1 + \left(\frac{[Ca^{2+}]_{JSR}}{0.4240}\right)^{15.24}}$$

$$Ac_{RyR,\infty}^{P} = 1.8891 \cdot 10^{-5} \cdot \left(\exp\left(\frac{[Ca^{2+}]_{JSR}}{1.4257}\right) - 1\right) + \frac{0.07}{1 + \left(\frac{0.003 \cdot \left(1 + 2.0 \cdot f_{RYR}^{P,CaMKII}\right)}{[Ca^{2+}]_{ss,CaL}}\right)^{8}}$$

$$Acl_{RyR,\infty}^{P} = 1.8891 \cdot 10^{-5} \cdot \left(\exp\left(\frac{[Ca^{2+}]_{JSR}}{1.4257}\right) - 1\right) + \frac{0.0005}{1 + \left(\frac{0.006 \cdot \left(1 + 2.0 \cdot f_{RYR}^{P,CaMKII}\right)}{[Ca^{2+}]_{ss,CaL}}\right)^{8}}$$

$$\tau_{RyR,Ac}^{P} = 0.3026 + 3.0000 \cdot \widehat{M}_{RyR}^{P}$$

$$k_{RyR,12}^{P} = \frac{Ac_{RyR,\infty}^{P}}{\tau_{RyR,Ac}^{P}}, \qquad k_{RyR,21}^{P} = \frac{1 - Ac_{RyR,\infty}^{P}}{\tau_{RyR,Ac}^{P}}$$

$$k_{RyR,43}^{P} = \frac{Acl_{RyR,\infty}^{P}}{\tau_{RyR,Ac}^{P}}, \qquad k_{RyR,34}^{P} = \frac{1 - Acl_{RyR,\infty}^{P}}{\tau_{RyR,Ac}^{P}}$$

$$k_{RyR,14,23}^{P} = \frac{\widehat{M}_{RyR}^{P}}{0.6000}, \qquad k_{RyR,41}^{P} = \frac{1}{409.16}$$

$$k_{RyR,32}^{P} = \frac{k_{RyR,41}^{P} \cdot k_{RyR,12}^{P} \cdot k_{RyR,34}^{P}}{k_{RyR,43}^{P} \cdot k_{RyR,21}^{P}}$$

$$\frac{dCB_{RyR}^{P}}{dt} = k_{RyR,21}^{P} \cdot OB_{RyR}^{P} + k_{RyR,41}^{P} \cdot CU_{RyR}^{P} - \left(k_{RyR,12}^{P} + k_{RyR,14,23}^{P}\right) \cdot CB_{RyR}^{P}$$

$$\frac{dOB_{RyR}^{P}}{dt} = k_{RyR,12}^{P} \cdot CB_{RyR}^{P} + k_{RyR,32}^{P} \cdot OU_{RyR}^{P} - \left(k_{RyR,21}^{P} + k_{RyR,14,23}^{P}\right) \cdot OB_{RyR}^{P}$$

$$\frac{dOU_{RyR}^{P}}{dt} = k_{RyR,14,23}^{P} \cdot OB_{RyR}^{P} + k_{RyR,43}^{P} \cdot CU_{RyR}^{P} - \left(k_{RyR,32}^{P} + k_{RyR,34}^{P}\right) \cdot OU_{RyR}^{P}$$

$$\frac{dCU_{RyR}^{P}}{dt} = k_{RyR,14,23}^{P} \cdot CB_{RyR}^{P} + k_{RyR,34}^{P} \cdot OU_{RyR}^{P} - \left(k_{RyR,41}^{P} + k_{RyR,43}^{P}\right) \cdot CU_{RyR}^{P}$$

$$I_{Rel}^{P} = \frac{3.5289 \cdot 10^{-5}}{V_{ss}} \cdot \left(OB_{RyR}^{P} + OU_{RyR}^{P}\right) \cdot \left([Ca^{2+}]_{JSR} - [Ca^{2+}]_{ss,CaL}\right)$$

$$I_{Rel} = \left(1 - f_{RyR}^{P,PKA}\right) \cdot I_{Rel}^{NP} + f_{RyR}^{P,PKA} \cdot I_{Rel}^{P}$$

1.4 Additional alterations in model parameters

Parameters of the non-phosphorylated and phosphorylated I_{CaL} formulations were adjusted slightly compared to Heijman et al. [6] to reduce the sensitivity of the model for the generation of early afterdepolarizations (EADs) in response to I_{Kr} blockade (requiring 80% I_{Kr} reduction to generate EADs compared to 25% in the absence of these changes) and to match the amount of action-potential duration (APD) prolongation after I_{Ks} blockade in the presence of adrenergic stimulation, while maintaining peak I-V and inactivation characteristics similar to experimental recordings.

$$ACT_{\infty}^{NP} = \frac{1}{\left(1 + \exp\left(-\frac{V_m - 13.56}{9.45}\right)\right) \cdot \left(1 + \exp\left(-\frac{V_m + 25}{5}\right)\right)}$$

$$I_{V,\tau}^{NP} = \frac{1}{\frac{1}{70.0 \cdot \left(1 + \exp\left((V_m + 49.10)/10.349\right)\right)} + \frac{1}{26.553 \cdot \left(1 + \exp(-(V_m + 0.213)/10.807)\right)}}$$

$$I_{V,\infty}^{NP} = \frac{1}{1.2474} \cdot \left(0.2474 + \frac{1}{1 + \exp\left((V_m + 19.0)/2.5\right)}\right)$$

$$Is_{V,\infty}^{NP} = \frac{1}{1.04} \cdot \left(0.04 + \frac{1}{1 + \exp\left((V_m + 19.0)/2.5\right)}\right)$$

$$ACT_{\infty}^{NP} = \frac{1}{\left(1 + \exp\left(-\frac{V_m - -4.798}{7.5699}\right)\right) \cdot \left(1 + \exp\left(-\frac{V_m + 25}{5}\right)\right)}$$

$$I^{P}_{V,\tau} = \frac{1}{\frac{1}{70.0 \cdot \left(1 + \exp\left((V_m + 49.10)/10.349\right)\right)} + \frac{1}{75 \cdot \left(1 + \exp(-(V_m + 0.213)/10.807)\right)}}$$

$$I^{P}_{V,\infty} = \frac{1}{1.02} \cdot \left(0.02 + \frac{1}{1 + \exp\left((V_m + 29.979)/3.1775\right)}\right)$$

$$Is^{P}_{V,\infty} = \frac{1}{1.0007} \cdot \left(0.0007 + \frac{1}{1 + \exp\left((V_m + 29.979)/3.1775\right)}\right)$$

2. Stochastic formulations of model components

2.1 Random number generation

The Mersenne-Twister random number generator [10] was used in all stochastic simulations to generate random variables from uniform and normal distributions. Binomial random variables $B(n, \mathrm{p})$ were generated using the Inverse Function Method or approximated from a normal distribution $N(n \cdot p, n \cdot p \cdot (1-p))$ when $\left|\frac{1}{\sqrt{n}} \cdot \left(\sqrt{\frac{1-p}{p}} - \sqrt{\frac{p}{1-p}}\right)\right| < 0.3$.

2.2 Stochastic formulations of Markov models

Stochastic simulations of Markov models of ion currents were performed as previously described for local control models [9,12,13]. Briefly, for a given state i in the Markov model with a state occupancy of n_i channels at time t, the probability for any channel to move to state j in the time interval $\Delta\mathrm{t}$ is given by $\mathrm{r}_{\mathrm{i,j}} \cdot \Delta\mathrm{t}$, where $\mathrm{r}_{\mathrm{i,j}}$ is the transition rate (in ms^{-1}) of the deterministic ODE model. Thus, the number of channels leaving state i in the interval $\Delta\mathrm{t}$ can be obtained from a multinomial distribution $M\left(n_i, \mathrm{r}_{\mathrm{i,1}} \cdot \Delta\mathrm{t}, \ldots, \mathrm{r}_{\mathrm{i,i-1}} \cdot \Delta\mathrm{t}, 1 - \Delta\mathrm{t} \cdot \sum \mathrm{r}_{\mathrm{i,j}}, \mathrm{r}_{\mathrm{i,i+1}} \cdot \Delta\mathrm{t}, \mathrm{r}_{\mathrm{ik}} \cdot \Delta\mathrm{t}\right)$. For sufficiently small $\Delta\mathrm{t}$, the transition probabilities are small and can be assumed to be independent. In this case, the number of channels leaving state i can be approximated based on k independent binomial distributions $B\left(n_i, \mathrm{r}_{\mathrm{i,j}} \cdot \Delta\mathrm{t}\right)$, significantly reducing the computational complexity [9]. The number of channels in state i is then updated at every time step by:

$$\mathrm{n_i}(\mathrm{t} + \Delta\mathrm{t}) = \mathrm{n_i}(\mathrm{t}) + \sum_{\mathrm{j \neq i}} B\left(\mathrm{n_j}(\mathrm{t}), \mathrm{r_{j,i}}(\mathrm{t}) \cdot \Delta\mathrm{t}\right) - \sum_{\mathrm{j \neq i}} B\left(\mathrm{n_i}(\mathrm{t}), \mathrm{r_{i,j}}(\mathrm{t}) \cdot \Delta\mathrm{t}\right)$$

This approach was employed for the Markov models of I_{CaL}, I_{Kr}, I_{Ks}, I_{Na} and RyR. In the case of simulations involving β-adrenergic stimulation, both the non-phosphorylated and phosphorylated channel populations [6] were simulated using this stochastic formulation.

2.3 Stochastic formulations of Hodgkin-Huxley-based models and instantaneous currents

Hodgkin-Huxley-based models are a subset of the class of Markov models with only independent transitions [14]. As such, the methodology for stochastic simulation of Markov models was also applied to the Markov representation of the equivalent Hodgkin-Huxley model for I_{To} and $I_{Cl(Ca)}$. For 'instantaneous' currents that are defined by an algebraic equation in the deterministic model, a simple 2-state (Open and Closed) model was used. The steady state distribution between the two states was defined by the original algebraic equation and a global time constant of 0.1 ms was used to determine the forward and backward rates between the two states. This approach was employed for stochastic simulations of I_{K1} and I_{Kur}.

2.4 Stochastic formulations of pumps and exchangers

In contrast to channels, where gating is a stochastic process but the flux of ions through an open channel is instantaneous, ion transport through pumps and exchangers is an active process. A single iteration of the pump or exchanger transports a given number of ions across the electrochemical gradient. As such, there is not a single 'open state' that defines the flux of ions but instead there is a state transition (or combination of transitions) that determines the flux of ions. Because of this, the throughput of a single pump is much lower than that of a channel. However, the expression of pumps is much higher than that of channels [15], thereby ensuring that an ionic balance can be maintained.

The SR and sarcolemmal Ca^{2+}-ATPases (underlying I_{up} and I_{pCa}, respectively) were simulated using the two-state, four-transition model structure of Tran et al. [16]. Parameters of the models were adjusted to reproduce the Ca^{2+} dependence of the deterministic formulations. We assumed that the state distribution changes more rapidly than the changes in ionic concentrations on both sides of the pump, resulting in a quasi-equilibrium situation in which individual rates are decoupled. The number of ions transported via each transition was derived from a binomial distribution and the net ion flux (or current) in the interval Δt was determined.

Equations for the stochastic I_{pCa} and I_{up} formulation are as follows:

$$k_{pCa,1+} = 5.2272 \cdot 10^3 \cdot [Ca^{2+}]_i \cdot \frac{1.00 \cdot 10^5}{\overline{N}_{pCa}}, \qquad k_{pCa,1-} = 1.7362 \cdot \frac{1.00 \cdot 10^5}{\overline{N}_{pCa}}$$

$$k_{pCa,2-} = 0.2503 \cdot \frac{1.00 \cdot 10^5}{\overline{N}_{pCa}}$$

$$n_{pCa} = \frac{k_{pCa,2-}}{k_{pCa,1+} + k_{pCa,1-} + k_{pCa,2-}} \cdot \overline{N}_{pCa}$$

$$I_{pCa} = \frac{B\left(n_{pCa}, k_{pCa,1+} \cdot \Delta t\right) \cdot z_{Ca} \cdot 10^9 \cdot F}{\Delta t \cdot N_A \cdot C_m \cdot A_{cap}}$$

$$f_{up}^{NP} = 1 - f_{up}^{PKA,only} - f_{up}^{CaMKII,only} - f_{up}^{Both}$$

$$k_{SERCA,1+}^{NP} = 4.6571 \cdot 10^3 \cdot [Ca^{2+}]_i \cdot \frac{2.00 \cdot 10^6}{\overline{N}_{SERCA}}, \qquad k_{SERCA,1-}^{NP} = 1.1618 \cdot \frac{2.00 \cdot 10^6}{\overline{N}_{SERCA}}$$

$$k_{SERCA,2+}^{NP} = 0.7514 \cdot [Ca^{2+}]_{NSR} \cdot \frac{2.00 \cdot 10^6}{\overline{N}_{SERCA}}, \qquad k_{SERCA,2-}^{NP} = 2.5205 \cdot \frac{2.00 \cdot 10^6}{\overline{N}_{SERCA}}$$

$$n_{SERCA}^{NP} = \frac{k_{SERCA,2+}^{NP} + k_{SERCA,2-}^{NP}}{k_{SERCA,1+}^{NP} + k_{SERCA,1-}^{NP} + k_{SERCA,2+}^{NP} + k_{SERCA,2-}^{NP}} \cdot f_{up}^{NP} \cdot \overline{N}_{SERCA}$$

$$J_{up}^{NP} = B\left(n_{SERCA}^{NP}, k_{SERCA,1+}^{NP} \cdot \Delta t\right) - B\left(f_{up}^{NP} \cdot \overline{N}_{SERCA} - n_{SERCA}^{NP}, k_{SERCA,2+}^{NP} \cdot \Delta t\right)$$

$$k_{SERCA,1+}^{P} = 7.5269 \cdot 10^3 \cdot [Ca^{2+}]_i \cdot \frac{2.00 \cdot 10^6}{\overline{N}_{SERCA}}, \qquad k_{SERCA,1-}^{P} = 0.7188 \cdot \frac{2.00 \cdot 10^6}{\overline{N}_{SERCA}}$$

$$k_{SERCA,2+}^{P} = 0.8456 \cdot [Ca^{2+}]_{NSR} \cdot \frac{2.00 \cdot 10^6}{\overline{N}_{SERCA}}, \qquad k_{SERCA,2-}^{P} = 2.6131 \cdot \frac{2.00 \cdot 10^6}{\overline{N}_{SERCA}}$$

$$n_{SERCA}^{P} = \frac{k_{SERCA,2+}^{P} + k_{SERCA,2-}^{P}}{k_{SERCA,1+}^{P} + k_{SERCA,1-}^{P} + k_{SERCA,2+}^{P} + k_{SERCA,2-}^{P}} \cdot \left(f_{up}^{PKA,only} + f_{up}^{Both}\right) \cdot \overline{N}_{SERCA}$$

$$J_{up}^{PKA} = B\left(n_{SERCA}^{P}, k_{SERCA,1+}^{P} \cdot \Delta t\right) - B\left(\left(f_{up}^{PKA,only} + f_{up}^{Both}\right) \cdot \overline{N}_{SERCA} - n_{SERCA}^{P}, k_{SERCA,2+}^{P} \cdot \Delta t\right)$$

$$k_{SERCA,1+}^{CaMKIIP} = 5.6542 \cdot 10^3 \cdot [Ca^{2+}]_i \cdot \frac{2.00 \cdot 10^6}{\bar{N}_{SERCA}}, \qquad k_{SERCA,1-}^{CaMKIIP} = 0.7009 \cdot \frac{2.00 \cdot 10^6}{\bar{N}_{SERCA}}$$

$$k_{SERCA,2+}^{CaMKIIP} = 1.1779 \cdot [Ca^{2+}]_{NSR} \cdot \frac{2.00 \cdot 10^6}{\bar{N}_{SERCA}}, \qquad k_{SERCA,2-}^{CaMKIIP} = 2.6034 \cdot \frac{2.00 \cdot 10^6}{\bar{N}_{SERCA}}$$

$$n_{SERCA}^{CaMKIIP} = \frac{k_{SERCA,2+}^{CaMKIIP} + k_{SERCA,2-}^{CaMKIIP}}{k_{SERCA,1+}^{CaMKIIP} + k_{SERCA,1-}^{CaMKIIP} + k_{SERCA,2+}^{CaMKIIP} + k_{SERCA,2-}^{CaMKIIP}} \cdot f_{up}^{CaMKII,only} \cdot \bar{N}_{SERCA}$$

$$J_{up}^{CaMKII} = B\left(n_{SERCA}^{CaMKIIP}, k_{SERCA,1+}^{CaMKIIP} \cdot \Delta t\right) - B\left(f_{up}^{CaMKII,only} \cdot \bar{N}_{SERCA} - n_{SERCA}^{CaMKIIP}, k_{SERCA,2+}^{CaMKIIP} \cdot \Delta t\right)$$

$$I_{up} = \left(1 + 2.25 \cdot f_{SERCA2a}^{CaMKII}\right) \cdot \frac{\left(J_{up}^{NP} + J_{up}^{PKA} + J_{up}^{CaMKII}\right) \cdot 10^9}{\Delta t \cdot N_A \cdot V_{NSR}}$$

where N_A equals Avogadro's number, $\bar{N}_x$ indicates the total number of channels of type x (see section 2.5) and all other constants are as previously defined [6].

Smith and Crampin have previously described a detailed model of the Na^+-K^+-ATPase based on a consecutive ("ping-pong") model for Na^+ and K^+ binding [17]. In their model, 'slippage' of ions is negligible due to a tight coupling between conformational changes of the pump and ion binding, thereby ensuring a strict 3 Na^+ : 2 K^+ stoichiometry. Based on these assumptions and since both pumps belong to the family of P-type cation transporters, [17] we employed a similar 2-state model structure as that used for SERCA. We set the 'backward' (counter-clockwise) rates to zero to ensure the strict stoichiometry. Forward rates were fitted to reproduce Na^+, K^+ and V_m dependence of the original I_{NaK} formulation for both the non-phosphorylated and phosphorylated populations:

$$\varphi_{NaK} = \frac{1}{1 + \exp\left(-(V_m + 92) \cdot \frac{F}{R \cdot T}\right)}$$

$$k_{NaK,1+}^{NP} = 1.1893 \cdot \varphi_{NaK} \cdot \left(\frac{[Na^+]_i}{11.5767 + [Na^+]_i}\right)^{1.5298} \cdot \frac{2.00 \cdot 10^6}{\bar{N}_{NaK}}, \qquad k_{NaK,1-}^{NP} = 0$$

$$k_{NaK,2+}^{NP} = 1.6425 \cdot \varphi_{NaK} \cdot \left(\frac{[K^+]_o}{2.3694 + [K^+]_o}\right)^{1.5298} \cdot \frac{2.00 \cdot 10^6}{\bar{N}_{NaK}}, \qquad k_{NaK,2-}^{NP} = 0$$

$$n_{NaK}^{NP} = \frac{k_{NaK,2+}^{NP} + k_{NaK,2-}^{NP}}{k_{NaK,1+}^{NP} + k_{NaK,1-}^{NP} + k_{NaK,2+}^{NP} + k_{NaK,2-}^{NP}} \cdot \left(1 - f_{INaK}^{P}\right) \cdot \bar{N}_{NaK}$$

$$F_{NaK}^{NP} = 3 \cdot B\left(n_{NaK}^{NP}, k_{NaK,1+}^{NP} \cdot \Delta t\right) - 2 \cdot B\left(\left(1 - f_{INaK}^{P}\right) \cdot \bar{N}_{NaK} - n_{NaK}^{NP}, k_{NaK,2+}^{NP} \cdot \Delta t\right)$$

$$k_{NaK,1+}^{P} = 1.1112 \cdot \varphi_{NaK} \cdot \left(\frac{[Na^+]_i}{7.6962 + [Na^+]_i}\right)^{1.5206} \cdot \frac{2.00 \cdot 10^6}{\bar{N}_{NaK}}, \qquad k_{NaK,1-}^{P} = 0$$

$$k_{NaK,2+}^{P} = 1.7324 \cdot \varphi_{NaK} \cdot \left(\frac{[K^+]_o}{2.3694 + [K^+]_o}\right)^{1.5206} \cdot \frac{2.00 \cdot 10^6}{\bar{N}_{NaK}}, \qquad k_{NaK,2-}^{P} = 0$$

$$n_{NaK}^{P} = \frac{k_{NaK,2+}^{P} + k_{NaK,2-}^{P}}{k_{NaK,1+}^{P} + k_{NaK,1-}^{P} + k_{NaK,2+}^{P} + k_{NaK,2-}^{P}} \cdot f_{INaK}^{P} \cdot \bar{N}_{NaK}$$

$$F_{NaK}^{P} = 3 \cdot B\left(n_{NaK}^{P}, k_{NaK,1+}^{P} \cdot \Delta t\right) - 2 \cdot B\left(f_{INaK}^{P} \cdot \bar{N}_{NaK} - n_{NaK}^{P}, k_{NaK,2+}^{P} \cdot \Delta t\right)$$

$$I_{NaK} = \frac{\left(F_{NaK}^{NP} + F_{NaK}^{P}\right) \cdot 10^9 \cdot F}{\Delta t \cdot N_A \cdot C_m \cdot A_{cap}}$$

Similar to the approach for the other transporters, we used the two-state, four-transition model structure of Tran et al. [16] for the Na^+/Ca^{2+} exchanger. Parameters of the model were adjusted to reproduce the intracellular and extracellular dependence on Na^+ and Ca^{2+}, as well as the V_m dependence of the deterministic I_{NaCa} formulation. As in the deterministic model, 80% of the exchangers were located in the bulk myoplasm, whereas 20% were located in the SR subspace. We assumed that the state distribution changes more rapidly than the changes in ionic concentrations on both sides of the pump, resulting in a quasi-equilibrium situation in which individual rates are decoupled. The model assumes a fixed stoichiometry of Na^+ and Ca^{2+} ions. As such, the number of charges transported in each direction was derived from a binomial distribution and the net ion flux (or current) in the interval Δt was determined as the difference between both directions:

$$f_V = \frac{1}{\exp\left(\eta \cdot V_m \cdot \frac{F}{R \cdot T}\right) + k_{sat} \cdot \exp\left(V_m \cdot \frac{F}{R \cdot T}\right)} \cdot \frac{1.0 \cdot 10^7}{\bar{N}_{NaCa}}$$

Exchangers located in the bulk myoplasm:

$$Ca_{act,i} = \frac{1}{1 + \left(\frac{K_{mCa,act}}{[Ca^{2+}]_i}\right)}$$

$$k_{NaCa,i}^{1+} = f_V \cdot Ca_{act,i} \cdot \left(-1.2231 \cdot 10^{-4} + \frac{17.472}{1 + \frac{8.4839 \cdot 10^{-2}}{[Ca^{2+}]_i}}\right) \cdot \left(1.7804 \cdot 10^{-1} + \frac{0.4006}{1 + \left(\frac{93.662}{[Na^+]_o}\right)^3}\right)$$

$$k_{NaCa,i}^{2+} = f_V \cdot Ca_{act,i} \cdot \left(2.7743 \cdot 10^{-1} + \frac{20.744}{1 + \frac{55.181}{[Ca^{2+}]_o}}\right) \cdot \left(1.0179 \cdot 10^{-2} + \frac{21.291}{1 + \left(\frac{43.388}{[Na^+]_i}\right)^3}\right)$$

$$k_{NaCa,i}^{1-} = f_V \cdot Ca_{act,i} \cdot 3.2353 \cdot 10^{-1}$$

$$k_{NaCa,i}^{2-} = f_V \cdot Ca_{act,i} \cdot 8.4790 \cdot 10^{-2} \cdot \exp\left(1.0825 \cdot V_m \cdot \frac{F}{R \cdot T}\right)$$

$$n_{NaCa,i} = \frac{k_{NaCa,i}^{2+} + k_{NaCa,i}^{1-}}{k_{NaCa,i}^{1-} + k_{NaCa,i}^{1+} + k_{NaCa,i}^{2-} + k_{NaCa,i}^{2+}} \cdot 0.8 \cdot \overline{N}_{NaCa}$$

$$F_{NaCa,i} = B\left(0.8 \cdot \overline{N}_{NaCa} - n_{NaCa,i}, k_{NaCa,i}^{2+} \cdot \Delta t\right) - B\left(n_{NaCa,i}, k_{NaCa,i}^{1+} \cdot \Delta t\right)$$

$$I_{NaCa,i} = \frac{F_{NaCa,i} \cdot 10^9 \cdot F}{\Delta t \cdot N_A \cdot C_m \cdot A_{cap}}$$

Exchangers located in the SR subspace:

$$Ca_{act,ss} = \frac{1}{1 + \left(\frac{K_{mCa,act}}{[Ca^{2+}]_{ss}}\right)}$$

$$k_{NaCa,ss}^{1+} = f_V \cdot Ca_{act,ss} \cdot \left(-1.2231 \cdot 10^{-4} + \frac{17.472}{1 + \frac{8.4839 \cdot 10^{-2}}{[Ca^{2+}]_{ss}}}\right) \cdot \left(1.7804 \cdot 10^{-1} + \frac{0.4006}{1 + \left(\frac{93.662}{[Na^+]_o}\right)^3}\right)$$

$$k_{NaCa,ss}^{2+} = f_V \cdot Ca_{act,ss} \cdot \left(2.7743 \cdot 10^{-1} + \frac{20.744}{1 + \frac{55.181}{[Ca^{2+}]_o}}\right) \cdot \left(1.0179 \cdot 10^{-2} + \frac{21.291}{1 + \left(\frac{43.388}{[Na^+]_{ss}}\right)^3}\right)$$

$$k_{NaCa,ss}^{1-} = f_V \cdot Ca_{act,ss} \cdot 3.2353 \cdot 10^{-1}$$

$$k_{NaCa,ss}^{2-} = f_V \cdot Ca_{act,ss} \cdot 8.4790 \cdot 10^{-2} \cdot \exp\left(1.0825 \cdot V_m \cdot \frac{F}{R \cdot T}\right)$$

$$n_{NaCa,ss} = \frac{k^{2+}_{NaCa,ss} + k^{1-}_{NaCa,ss}}{k^{1-}_{NaCa,ss} + k^{1+}_{NaCa,ss} + k^{2-}_{NaCa,ss} + k^{2+}_{NaCa,ss}} \cdot 0.2 \cdot \overline{N}_{NaCa}$$

$$F_{NaCa,ss} = B\left(0.2 \cdot \overline{N}_{NaCa} - n_{NaCa,ss}, k^{2+}_{NaCa,ss} \cdot \Delta t\right) - B\left(n_{NaCa,ss}, k^{1+}_{NaCa,ss} \cdot \Delta t\right)$$

$$I_{NaCa,ss} = \frac{F_{NaCa,ss} \cdot 10^9 \cdot F}{\Delta t \cdot N_A \cdot C_m \cdot A_{cap}}$$

With $K_{mCa,act} = 1.2400 \cdot 10^{-4} \frac{mmol}{L}, k_{sat} = 0.32\ and\ \eta = 0.27$, as previously defined [6].

2.5 Number of channels / transporters

The appropriate number of channels, pumps or exchangers of each type was estimated based on the whole-cell conductance in the model and experimentally-determined single-channel conductance (**Table S1**). It should be noted that $\overline{N}_x$ represents the maximum number of effective channels that can open (i.e., the number of channels open when open probability equals 1.0). There may be additional channels in a myocyte that do not open. In particular, if the deterministic formulation of a current x is adjusted such that the open probability is halved and the whole-cell conductance is doubled (giving the same net current), then $\overline{N}_x$ as given below would be increased by a factor of 2.0. However, because open probability is reduced, the number of channels that participate during the AP would not change.

3. Generation of a distribution of single-cell models

We employed the method previously described by Sarkar and Sobie [32] to generate a distribution of individual single-cell models. Briefly, 13 random numbers were generated from a Gaussian distribution with mean 1.0 and standard deviation 0.3. The whole-cell conductance of each stochastic current was then multiplied by one of the random numbers and the deterministic model was simulated with that parameter set under the given conditions until APD and intracellular concentrations reached steady-state (2000 seconds of simulation). The state vector was stored and used as initial state for stochastic simulations with the same parameter set. The number of channels (Section 2.5) was not altered in these stochastic simulations. APD and BVR characteristics were stored together with each parameter set and employed in Figures 3 and 8.

4. Experimental recordings in isolated canine ventricular myocytes

This investigation conformed with the Guide for the Care and Use of Laboratory Animals published by the US National Institutes of Health (NIH Publication No. 85-23, revised 1996). Animal handling was in accordance with the European Directive for the Protection of Vertebrate Animals Used for Experimental and Other Scientific Purposes (86/609/EU).

Transmembrane action potentials (APs) were recorded from isolated left-ventricular canine midmyocardial myocytes as previously described [33,34]. Briefly, the left anterior descending coronary artery was cannulated and perfused. After ~20 min of collagenase perfusion and subsequent washout of the enzyme, the epicardial surface layer was removed from the LV wedge until a depth of ≥3 mm was reached. Softened tissue samples were collected from the midmyocardial layer underneath while contamination with the endocardium was avoided. Samples were gently agitated, filtered and washed. Cells were stored at room temperature in standard buffer solution. Only quiescent rod-shaped cells with clear cross-striations were used for the experiments within 48 h of isolation. APs were recorded at 37 °C using high-resistance (30–60 MΩ) glass microelectrodes filled with 3 M KCl with a microelectrode amplifier (Axoclamp-2B, Axon Instruments, Inc). Intracellular pacing was applied at various cycle lengths (CLs; 500 ms – 2000 ms). Only cells showing a stable spike-and-dome AP morphology and resting membrane potential were accepted for the experiments.

References

1. Silva J, Rudy Y (2005) Subunit interaction determines I_{Ks} participation in cardiac repolarization and repolarization reserve. Circulation 112: 1384-1391.
2. Liu DW, Antzelevitch C (1995) Characteristics of the delayed rectifier current (I_{Kr} and I_{Ks}) in canine ventricular epicardial, midmyocardial, and endocardial myocytes. A weaker I_{Ks} contributes to the longer action potential of the M cell. Circ Res 76: 351-365.
3. Yang T, Snyders DJ, Roden DM (1995) Ibutilide, a methanesulfonanilide antiarrhythmic, is a potent blocker of the rapidly activating delayed rectifier K^+ current (I_{Kr}) in AT-1 cells. Concentration-, time-, voltage-, and use-dependent effects. Circulation 91: 1799-1806.
4. Jurkiewicz NK, Sanguinetti MC (1993) Rate-dependent prolongation of cardiac action potentials by a methanesulfonanilide class III antiarrhythmic agent. Specific block of rapidly activating delayed rectifier K^+ current by dofetilide. Circ Res 72: 75-83.
5. Clancy CE, Rudy Y (2002) Na^+ channel mutation that causes both Brugada and long-QT syndrome phenotypes: a simulation study of mechanism. Circulation 105: 1208-1213.
6. Heijman J, Volders PGA, Westra RL, Rudy Y (2011) Local control of β-adrenergic stimulation: Effects on ventricular myocyte electrophysiology and Ca^{2+}-transient. J Mol Cell Cardiol 50: 863-871.
7. Baba S, Dun W, Boyden PA (2004) Can PKA activators rescue Na^+ channel function in epicardial border zone cells that survive in the infarcted canine heart? Cardiovasc Res 64: 260-267.
8. Livshitz LM, Rudy Y (2007) Regulation of Ca^{2+} and electrical alternans in cardiac myocytes: role of CAMKII and repolarizing currents. Am J Physiol Heart Circ Physiol 292: H2854-2866.
9. Restrepo JG, Weiss JN, Karma A (2008) Calsequestrin-mediated mechanism for cellular calcium transient alternans. Biophys J 95: 3767-3789.
10. Matsumoto M, Nishimura T (1998) Mersenne twister: a 623-dimensionally equidistributed uniform pseudo-random number generator. ACM Trans Model Comput Simul 8: 3-30.
11. Rao CV, Wolf DM, Arkin AP (2002) Control, exploitation and tolerance of intracellular noise. Nature 420: 231-237.
12. Tanskanen AJ, Greenstein JL, O'Rourke B, Winslow RL (2005) The role of stochastic and modal gating of cardiac L-type Ca^{2+} channels on early after-depolarizations. Biophys J 88: 85-95.
13. Gaur N, Rudy Y (2011) Multiscale modeling of calcium cycling in cardiac ventricular myocyte: macroscopic consequences of microscopic dyadic function. Biophys J 100: 2904-2912.
14. Rudy Y, Silva JR (2006) Computational biology in the study of cardiac ion channels and cell electrophysiology. Q Rev Biophys 39: 57-116.
15. Hille B (2001) Ion channels of excitable membranes. Sunderland, Mass.: Sinauer. xviii, 814 p. p.
16. Tran K, Smith NP, Loiselle DS, Crampin EJ (2009) A thermodynamic model of the cardiac sarcoplasmic/endoplasmic Ca^{2+} (SERCA) pump. Biophys J 96: 2029-2042.
17. Smith NP, Crampin EJ (2004) Development of models of active ion transport for whole-cell modelling: cardiac sodium-potassium pump as a case study. Prog Biophys Mol Biol 85: 387-405.
18. Guia A, Stern MD, Lakatta EG, Josephson IR (2001) Ion concentration-dependence of rat cardiac unitary L-type calcium channel conductance. Biophys J 80: 2742-2750.
19. Collier ML, Levesque PC, Kenyon JL, Hume JR (1996) Unitary Cl^- channels activated by cytoplasmic Ca^{2+} in canine ventricular myocytes. Circ Res 78: 936-944.

20. Kääb S, Nuss HB, Chiamvimonvat N, O'Rourke B, Pak PH, et al. (1996) Ionic mechanism of action potential prolongation in ventricular myocytes from dogs with pacing-induced heart failure. Circ Res 78: 262-273.
21. Liu GX, Zhou J, Nattel S, Koren G (2004) Single-channel recordings of a rapid delayed rectifier current in adult mouse ventricular myocytes: basic properties and effects of divalent cations. J Physiol 556: 401-413.
22. Veldkamp MW, van Ginneken AC, Bouman LN (1993) Single delayed rectifier channels in the membrane of rabbit ventricular myocytes. Circ Res 72: 865-878.
23. Yang Y, Sigworth FJ (1998) Single-channel properties of I_{Ks} potassium channels. J Gen Physiol 112: 665-678.
24. Romey G, Attali B, Chouabe C, Abitbol I, Guillemare E, et al. (1997) Molecular mechanism and functional significance of the MinK control of the KvLQT1 channel activity. J Biol Chem 272: 16713-16716.
25. Sesti F, Goldstein SA (1998) Single-channel characteristics of wild-type I_{Ks} channels and channels formed with two minK mutants that cause long QT syndrome. J Gen Physiol 112: 651-663.
26. Yue L, Feng J, Li GR, Nattel S (1996) Characterization of an ultrarapid delayed rectifier potassium channel involved in canine atrial repolarization. J Physiol 496: 647-662.
27. Sridhar A, da Cunha DN, Lacombe VA, Zhou Q, Fox JJ, et al. (2007) The plateau outward current in canine ventricle, sensitive to 4-aminopyridine, is a constitutive contributor to ventricular repolarization. Br J Pharmacol 152: 870-879.
28. Ono K, Fozzard HA, Hanck DA (1993) Mechanism of cAMP-dependent modulation of cardiac sodium channel current kinetics. Circ Res 72: 807-815.
29. Blaustein MP, Lederer WJ (1999) Sodium/calcium exchange: its physiological implications. Physiol Rev 79: 763-854.
30. Dobretsov M, Hastings SL, Stimers JR (1998) Na^+-K^+ pump cycle during β-adrenergic stimulation of adult rat cardiac myocytes. J Physiol 507: 527-539.
31. Fedida D, Giles WR (1991) Regional variations in action potentials and transient outward current in myocytes isolated from rabbit left ventricle. J Physiol 442: 191-209.
32. Sarkar AX, Sobie EA (2011) Quantification of repolarization reserve to understand interpatient variability in the response to proarrhythmic drugs: A computational analysis. Heart Rhythm 8: 1749-1755.
33. Volders PGA, Sipido KR, Carmeliet E, Spätjens RLHMG, Wellens HJ, et al. (1999) Repolarizing K^+ currents I_{TO1} and I_{Ks} are larger in right than left canine ventricular midmyocardium. Circulation 99: 206-210.
34. Johnson DM, Heijman J, Pollard CE, Valentin JP, Crijns HJ, et al. (2010) I_{Ks} restricts excessive beat-to-beat variability of repolarization during beta-adrenergic receptor stimulation. J Mol Cell Cardiol 48: 122-130.
35. Johnson DM, Heijman J, Bode EF, Greensmith DJ, Van der Linde H, et al. (2013) Diastolic spontaneous calcium release from the sarcoplasmic reticulum increases beat-to-beat variability of repolarization in canine ventricular myocytes after β-adrenergic stimulation. Circ Res 112: 246-256.
